# A Comparative Study of Short Linear Motif Compositions of the Influenza A Virus Ribonucleoproteins

**DOI:** 10.1371/journal.pone.0038637

**Published:** 2012-06-08

**Authors:** Chu-Wen Yang

**Affiliations:** Department of Microbiology, Soochow University, Shih-Lin, Taipei, Taiwan, Republic of China; University of Ottawa, Canada

## Abstract

Protein-protein interactions through short linear motifs (SLiMs) are an emerging concept that is different from interactions between globular domains. The SLiMs encode a functional interaction interface in a short (three to ten residues) poorly conserved sequence. This characteristic makes them much more likely to arise/disappear spontaneously via mutations, and they may be more evolutionarily labile than globular domains. The diversity of SLiM composition may provide functional diversity for a viral protein from different viral strains. This study is designed to determine the different SLiM compositions of ribonucleoproteins (RNPs) from influenza A viruses (IAVs) from different hosts and with different levels of virulence.

The 96 consensus sequences (regular expressions) of SLiMs from the ELM server were used to conduct a comprehensive analysis of the 52,513 IAV RNP sequences. The SLiM compositions of RNPs from IAVs from different hosts and with different levels of virulence were compared. The SLiM compositions of 845 RNPs from highly virulent/pandemic IAVs were also analyzed. In total, 292 highly conserved SLiMs were found in RNPs regardless of the IAV host range. These SLiMs may be basic motifs that are essential for the normal functions of RNPs. Moreover, several SLiMs that are rare in seasonal IAV RNPs but are present in RNPs from highly virulent/pandemic IAVs were identified.

The SLiMs identified in this study provide a useful resource for experimental virologists to study the interactions between IAV RNPs and host intracellular proteins. Moreover, the SLiM compositions of IAV RNPs also provide insights into signal transduction pathways and protein interaction networks with which IAV RNPs might be involved. Information about SLiMs might be useful for the development of anti-IAV drugs.

## Introduction

Protein-protein interactions can be categorized into the following four classes: domain-domain interactions, mutual fit interactions, induced fit interactions and linear motif-domain interactions [1]. The binding site for linear motif-domain interactions is a short peptide of only a few (three to ten) residues that is called a “short linear motif” (SLiM) [1]. Three characteristics differentiate SLiMs from globular domains. The first characteristic is the ability of SLiMs to encode a functional interaction interface in a short (three to ten residues) and often poorly conserved sequence. The short length of the motifs also makes them much more likely to arise/disappear spontaneously via mutations, which make them more evolutionarily labile (i.e. likely to appear *de novo* in unrelated protein sequences) [1]. The second feature of SLiMs is that the richness of potential motif-domain interactions is higher than the domain-domain interactions within a given length of sequence. The third characteristic of SLiMs is that because only a small number of residues are involved, the interactions tend to be transient and have low binding affinities. Therefore, they are well suited for mediating functions that require a fast response to changing stimuli, such as interactions between SH2 motifs (which binds a phosphorylated tyrosine) and phosphorylation sites on its binding partners. These three characteristics may provide a flexible molecular basis for fast evolved proteins of RNA viruses with great versatility.

Several pioneering studies were significant for the characterization of SLiMs in viral proteins. Davey et al. collected 52 experimentally validated SLiMs present in viral proteins [2]. These examples of viral SLiMs are present in highly studied viral proteins that are responsible for relevant diseases, such as cancers (human papillomavirus, Epstein-Barr virus, human T-cell lymphotropic virus and adenovirus), immunodeficiency (HIV) or the flu (influenza). Currently, a comprehensive SLiM database has been established that is called the Eukaryotic Linear Motif (ELM) Resource for Functional Sites in Proteins [3]. Based on the motif patterns provided in the ELM database, computational analysis can be performed to identify high potential SLiMs in target proteins and can reduce the arduous and high cost laboratory procedures that are required to identify them.

The ribonucleoprotein (RNP) complex of influenza A virus (IAV), which is composed of the PA, PB1, PB2 and NP proteins, is essential for virus replication in cells. The RNP complex replicates the segments of the RNA virus genome and transcribes its genes [4]. Moreover, the RNP complex affects the evolution of IAV through its error-prone RNA polymerase, which produces variants of the viral proteins, including the HA, NA and the RNP themselves. Therefore, virus strains that are better adapted to a new host species are created [5]. Additionally, the RNP complex represents a promising drug target because its activities are distinct from RNA polymerase found in the host cell [6]. However, despite its biomedical importance, the absence of detailed SLiM information of the RNPs has limited our mechanistic understanding of RNP functions and the ability to design better drugs.

The present study sought to gain a deeper understanding of IAV RNP-host interactions that affect RNP activity in human cells. Using a functional proteomics approach, 96 SLiM consensus sequences (regular expressions) from the ELM server [3] were used to perform a systemic and comprehensive analysis of IAV RNPs. A comparative study of the SLiM composition of RNPs from IAVs from different hosts and highly virulent/pandemic (HP) IAV strains was performed. Several SLiMs, including highly conserved SLiMs, IAV host specific SLiMs and/or HP IAV specific SLiMs, that might affect RNP function were identified. The results of this study not only provide information on the SLiM compositions of IAV RNPs but also provide insights into the signal transduction pathways and protein interaction networks which IAV RNPs might be involved in.

## Materials and Methods

### Data

In total 63,237 sequences from IAV RNPs were retrieved from the NCBI Influenza Database. After checking for completeness by assessing the N-terminus and the length, 52,505 IAV RNP sequences were used in this study. This data set includes 18,952, 29,230 and 4,323 RNP sequences from IAVs from avian, human and mammalian hosts, respectively (Information S1). Hosts of the avian and mammalian IAVs are listed in Information S2. A set of 845 RNP sequences (Information S3) from highly virulent/pandemic (HP) IAVs, including the 1918 H1N1 IAV from the “Spanish Flu”, the H2N2 IAV from the 1957 outbreak, the H3N2 IAV from the 1957 outbreak, the H1N1 IAV from the 1977 Russia outbreak, the 2009 H1N1 IAV from the “swine flu”, the H5N1 IAV from the 1997 Hong Kong outbreak and the 2004–2008 highly pathogenic H5N1IAVs from Vietnam, Indonesia and Thailand were analysed.

Information regarding the SLiMs was retrieved from the ELM server (the Eukaryotic Linear Motif Resource for Functional Sites in Proteins) [3]. SLiMs were classified into four types: protease cleavage sites (prefix CLV), protein motif interacting/binding sites (prefix LIG), posttranslational modification sites (prefix MOD) and subcellular targeting signals (prefix TRG) [3]. In total, 96 SLiMs that are each supported by more than five real sequences were used in this study and are listed in Information S4.

### Statistical Methods

The tests for differences among *k* proportions were performed as follows [7]:
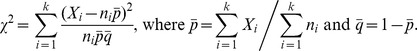

*i* = 1, 2, …, *k*. The degree of freedom *ν* = *k*−1.

The log-likelihood ratio tests for independence were performed as follows [7]:
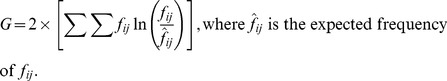
The degree of freedom *ν* = (*i*−1)(*j*−1).

The Shannon entropy H was introduced by Shannon as a measurement of uncertainty [8]. This method has been applied to measure the diversity of amino acids to identify biologically important amino acids in viral proteins from Papillomavirus [9], West Nile virus [10], HCV [11] and IAV [12], [13], [14]. The Shannon diversity index of each SLiM was computed by the formula:


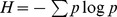
, where *p* is the proportion of each SLiM [7].

#### Identity Distributions of Pairwise Alignments

For a given a SLiM, all sequences harbor the SLiM from each host class were used to perform pairwise alignments and to compute the identity of each pair. For example, the SLiM LIG_PTB_Apo_2_328 was identified in 4777, 4715 and 746 PA sequences from avian, human and mammalian IAVs, respectively. The 11,407,476 identities from (4777×(4777−1)/2) pairwise alignments were computed using PA sequences from avian IAVs. Similarly, the 11,113,255 identities from (4715×(4715−1)/2) pairwise alignments were computed using PA sequences from human IAVs. The 277,885 identities from (746×(746−1)/2) pairwise alignments were computed using PA sequences from mammalian IAVs. Then, the distributions of the three sets of identities were plotted together.

### Perl Programming

The computer programs that were used in this study for data manipulation and pattern (regular expression) match were written by the author using the Perl programming language. The program used for this data analysis is available on request.

## Results

### An overview of the motif-based diversity of IAV RNP sequences

**Figure 1:**
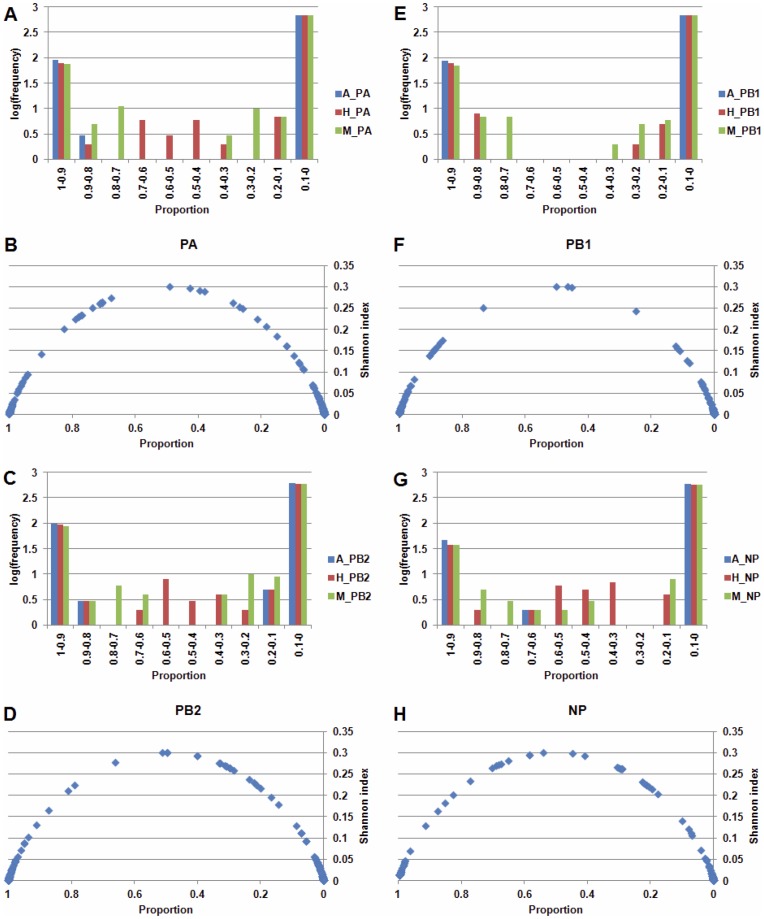
The distribution of occurrence and the Shannon diversity index of SLiMs in IAV RNPs. For A, C, E and G the Y-axis indicates the number of identified SLiMs, and the X-axis indicates the occurrence of the SLiMs. For B, D, F and H the Y-axis indicates the Shannon diversity index. The X-axis indicates the occurrences of SLiMs. The occurrence of a SLiM at an aa position is computed by the number of the RNP sequences with the SLiM at the same position divided by total number of the RNP sequences. (A) The frequency distribution of the identified SLiMs in the PA protein sequences. (B) The Shannon diversity index distribution of the identified SLiMs in the PA protein sequences. (C) The frequency distribution of the identified SLiMs in the PB1 protein sequences. (D) The Shannon diversity index distribution of the identified SLiMs in the PB1 protein sequences. (E) The frequency distribution of the identified SLiMs in the PB2 protein sequences. (F) The Shannon diversity index distribution of the identified SLiMs in the PB2 protein sequences. (G) The frequency distribution of the identified SLiMs in the NP protein sequences. (H) The Shannon diversity index distribution of the identified SLiMs in the NP protein sequences.

In total, 96 SLiM consensus sequences (regular expressions) were retrieved from the ELM server and were used to analyze the diversity of SLiM compositions for 52,505 IAV RNP sequences (Information S1). For each RNP, the occurrence of a SLiM at a position in the RNP is computed by the number of the RNP sequences with the SLiM at a given position divided by total number of the RNP sequences. For example, 7,222 PA protein sequences from human IAVs were used in this study. A SLiM with an occurrence of 1% for the PA protein from human IAVs means that 72 of the 7,222 PA protein sequences from human IAVs have the SLiM at the same position. As shown in Figure 1A, 1C, 1E and 1G, the identified SLiMs can be divided into the following three categories: an occurrence of greater than 90%, an occurrence between 90–10% and an occurrence of less than 10%. The group of SLiMs with an occurrence of over 90% (highly conserved) may be basic functional motifs for each RNP. A small fraction of the SLiMs with an occurrence between 90–10% forms the second group which represents partially conserved motifs (conserved in a subset of a RNP). SLiMs of this group have higher Shannon diversity indices than those from the other groups for all four RNPs (Figure 1B, 1D, 1F and 1H). In contrast, most of SLiMs belong to the third group, which occur in less than 10% of the RNP. These results indicate that most SLiMs might be created sporadically by mutations and might be present in specific IAV strains. Together, the combination of occurrences and the Shannon diversity index can be used to distinguish different types of diversity of the SLiM composition. As shown in Figure 1, the first group of SLiMs has low Shannon diversity index value and high occurrence (greater than 90%), which represents highly conserved motifs (common for all IAVs). The second group of SLiM has high Shannon diversity index value and occurrence of 90–10%, which represents partially conserved motifs. However, the number of SLiMs in this group is few (Table 1). In contrast, the third group of SLiM has both low Shannon diversity index value and low occurrence (less than 10%). The number of SLiMs in this group is plenty (Figure 1 and Table 1). The average numbers of SLiMs per gene (numbers in the brackets beside the raw frequency in Table 1) indicate the second and third SLiM groups represent different types of SLiM composition diversity.

**Table 1:** A Comparison of the SLiM distributions of RNPs from highly virulent/pandemic (Pan) IAVs and RNPs from all IAVs. Numbers in the brackets beside the raw frequency are average numbers of SLiMs per gene.

Occurrence	1∼0.9	0.9∼0.1	0.1∼0
All PA	80 (79.3)	24 (12.3)	687 (1.2)
Pan_PA	80 (79.0)	24 (13.6)	48 (3.3)
All PB1	80 (79.3)	14 (8.0)	689 (0.9)
Pan_PB1	80 (79.0)	13 (8.8)	33 (0.7)
All PB2	94 (93.3)	25 (9.7)	593 (1.2)
Pan_PB2	94 (92.5)	23 (9.6)	40 (2.2)
All NP	37 (36.1)	28 (12.5)	565 (0.7)
Pan_NP	37 (36.9)	25 (11.0)	21 (0.2)

#### Comparison of PA protein SLiM compositions among IAVs from different hosts

To gain a deeper understanding of the SLiM composition of IAV RNPs, the SLiM compositions of IAV RNPs from different hosts were compared. Using the PA protein as an example, the comparison of the SLiM composition of PA proteins among IAVs from avian (A_PA), human (H_PA) and mammalian (M_PA) hosts reveals that the 791 identified SLiMs can be classified into three groups (Information S5). The first group is composed of 80 highly conserved SLiMs (with an occurrence of greater than 90% in all PA protein sequences) that are common in all PA proteins regardless of the IAV host range (Information S6). The 80 SLiMs may be basic motifs that are essential for normal PA protein functions. The second group includes 24 partially conserved SLiMs (with an occurrence between 90–10% for all PA protein sequences). The third group contains 687 low occurrence SLiMs (with an occurrence of less than 10% in all PA protein sequences). 21 locations that contain two or more overlapping SLiMs from the first group were found (red rectangles in Information S6). Locations with highly conserved overlapping SLiMs may represent short protein domains that can respond to multiple host factors/pathways (see discussion).

**Figure 2:**
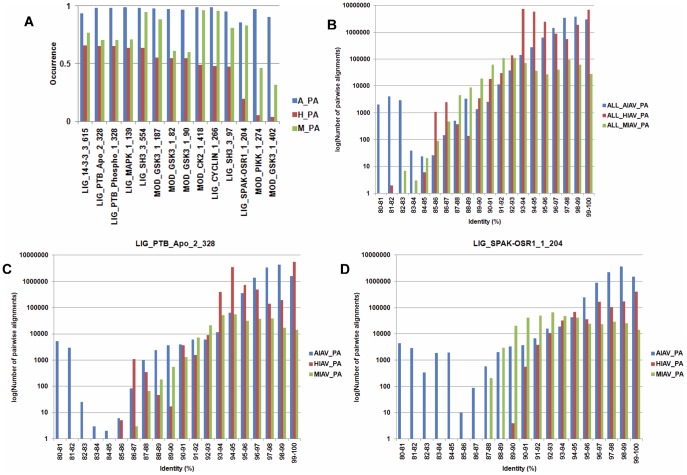
SLiMs from IAV PA proteins that have a differential occurrence in IAVs from different hosts. (A) 14 SLiMs that have a differential occurrence in IAV PA proteins from different hosts. A_PA, H_PA and M_PA indicate the PA proteins from avian, human and IAV, respectively. The Y-axis indicates the occurrence of each identified SLiM. The X-axis indicates the name and position of each identified SLiM in the PA proteins. For example, “LIG_14-3-3_3” in “LIG_14-3-3_3_615” is the name of the SLiM, and 615 is the amino acid position where the SLiM starts. (B) The distribution of pairwise alignment identity of all PA protein sequences from avian, human and mammalian IAVs. (C) The distribution of pairwise alignment identity of PA protein sequences which harbor the SLiM LIG_PTB_Apo_2_328 from avian, human and mammalian IAVs. (D) The distribution of pairwise alignment identity of PA protein sequences which harbor the SLiM LIG_SPAK-OSR1_1_204 from avian, human and mammalian IAVs. For B, C and D, the X-axis indicates the number of pairwise alignments of IAV PA protein sequences. The Y-axis indicates the identity of pairwise alignment (the percentage of identical amino acids that are the same in both PA sequences). Blue: PA protein sequences from avian IAVs. Red: PA protein sequences from human IAVs. Green: PA protein sequences from mammalian IAVs.

To uncover IAV host specific motifs in PA proteins in the second group, the test for difference among *k* proportions was performed. Because of the large sample size used in this study, a *p* value of 10^−100^ was used as the cut-off value. In total, 14 SLiMs that have a *p* value of less than 10^−100^ and have an occurrence of greater than 80% in the PA protein sequences from avian, human or mammalian IAVs were identified. Moreover, the log-likelihood ratio tests were performed to test the dependence between the existence of a SLiM and the host origin of the PA protein. All 14 SLiMs have a *p* value of less than 0.05 indicate there are dependences between the existence of the 14 SLiMs and the host origin of PA proteins. As shown in Figure 2A, all 14 SLiMs have a lower occurrence in PA proteins from human IAVs than in PA proteins from avian and mammalian IAVs. Notably, three of the SLiMs (LIG_SPAK-OSR1_1_204, MOD_PIKK_1_274 and MOD_GSK3_1_402) occur rarely in PA proteins from human IAVs. It is known that the PA sequences are not completely independent because there are phylogenetic relationships between them. A SLiM may be derived either from sequences of the same lineage (founder effect) or from host adaptation (convergent evolution). To reveal the underlying phylogenetic relationship, all PA sequences from each host class were used to perform pairwise alignments and the identities of all sequence pairs were computed (Figure 2B). Moreover, all sequences harbor a SLiM from each host class were used to perform pairwise alignments and the identities of all sequence pairs were computed. Two of the 14 SLiMs are shown in Figure 2C and 2D as examples. If two PA protein sequences with an identity greater than 95% are considered as sequences from the same lineage, then a SLiM identified from PA protein sequences with an identity greater than 95% may represent a result of founder effect. In contrast, a SLiM identified from PA protein sequences with an identity less than 95% may represent an event of host adaptation (convergent evolution). Results in Figure 2C and 2D suggest both of the founder effect and host adaptation were occurred. Similar phenomena were found for other SLiMs (Information S7).

### Comparison of the SLiM compositions of PA protein from IAVs with different virulence

To uncover potential IAV virulence-associated motifs in PA proteins, a comparison of PA SLiM compositions from all IAVs and HP IAVs was conducted. The 152 SLiMs identified can be classified into three groups (Information S8). The first group is composed of 80 highly conserved SLiMs (with an occurrence of greater than 90% in all PA protein sequences) that are common in all PA proteins regardless of IAV virulence. The second group includes 24 partially conserved SLiMs (with an occurrence between 90–10% in all PA protein sequences). The third group has 48 low occurrence SLiMs (with an occurrence of less than 10% in all PA protein sequences). Therefore, the number of candidate motifs in the third group was reduced from 687 to 48. If a SLiM appears in PA proteins from HP IAVs but is very rare in PA proteins from human IAVs, it may be associated with the virulence of HP IAVs through its effect on the function of the PA protein. Using two criteria, a very low occurrence (less than 10%) in human IAV PA proteins and its presence in HP IAV PA proteins, 47 SLiMs from the second (24 motifs) and third (48 motifs) groups were identified. Moreover, a SLiM from the second group that has a high occurrence in PA proteins from both avian and mammalian IAVs but a low occurrence (19.5%) in PA proteins from human IAVs was found. The 48 SLiMs (Information S9) are candidate sites that might affect PA protein activity and might be associated with IAV transcription and/or replication efficiency. 10 of the 48 SLiMs are even more notable. 3 of the 10 SLiMs (LIG_14-3-3_3_57, MOD_PIKK_1_650 and MOD_CK2_1_650) are likely avian IAV specific (labelled “A” in Information S9). 4 of the 10 SLiMs (MOD_CK2_1_17, LIG_FHA_2_18 and MOD_CK2_1_686) are likely mammalian IAV specific (labelled “M” in Information S9). Another 3 of the 10 SLiMs (LIG_SPAK-OSR1_1_204, MOD_PIKK_1_274 and MOD_GSK3_1_402) have a high occurrence in PA proteins from avian and mammalian IAVs (labelled “A & M” in Information S9).

### Comparison of the SLiM compositions of PB1 proteins among IAVs from different hosts

A comparison of PB1 SLiM compositions among IAVs from avian (A_PB1), human (H_PB1) and mammalian (M_PB1) hosts reveals that the 783 identified SLiMs can be classified into three groups (Information S10). The first class is composed of 81 highly conserved SLiMs (with an occurrence of greater than 90% in all PB1 protein sequences) that are common in all PB1 proteins regardless of the IAV host range (Information S11). These 81 SLiMs may be basic motifs that are essential for normal PB1 protein functions. The second class includes 13 partially conserved SLiMs (with an occurrence between 90–10% in all PB1 protein sequences). The third class has 689 low occurrence SLiMs (with an occurrence of less than 10% in all PB1 protein sequences). 17 locations that contain two or more overlapping SLiMs from the first group were identified (red rectangles in Information S11).

**Figure 3:**
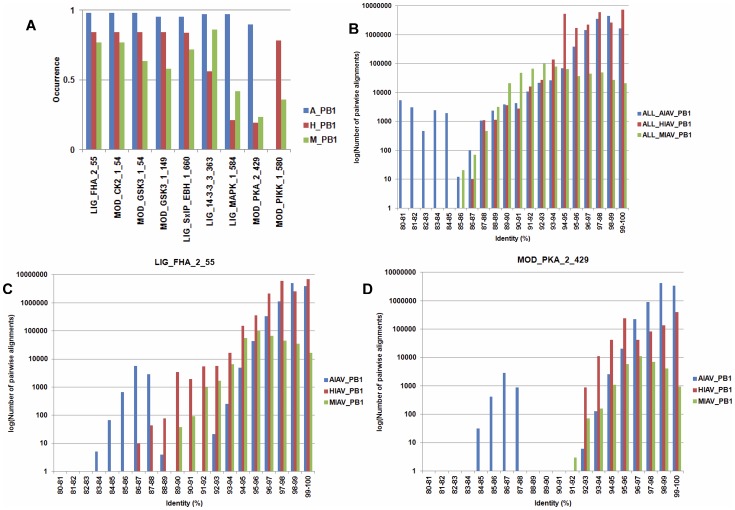
SLiMs from IAV PB1 proteins that have a differential occurrence in IAVs from different hosts. (A) 9 SLiMs that have a differential occurrence in IAV PB1 proteins from different hosts. A_PB1, H_PB1 and M_PB1 indicate the PB1 proteins from avian, human and IAV, respectively. The Y-axis indicates the occurrence of each identified SLiM. The X-axis indicates the name and position of each identified SLiM in the PB1 proteins. For example, “LIG_FHA_2” in “LIG_FHA_2_55” is the name of the SLiM, and 55 is the amino acid position where the SLiM starts. (B) The distribution of pairwise alignment identity of all PB1 protein sequences from avian, human and mammalian IAVs. (C) The distribution of pairwise alignment identity of PB1 protein sequences which harbor the SLiM LIG_FHA_2_55 from avian, human and mammalian IAVs. (D) The distribution of pairwise alignment identity of PB1 protein sequences which harbor the SLiM MOD_PKA_2_429 from avian, human and mammalian IAVs. For B, C and D, the X-axis indicates the number of pairwise alignments of IAV PB1 protein sequences. The Y-axis indicates the identity of pairwise alignment (the percentage of identical amino acids that are the same in both PB1 sequences). Blue: PB1 protein sequences from avian IAVs. Red: PB1 protein sequences from human IAVs. Green: PB1 protein sequences from mammalian IAVs.

To uncover IAV host specific motifs in PB1 proteins in the second group, the test for difference among *k* proportions is performed. Using the *p* value of 10^−100^ as a cut off value, 9 SLiMs were identified that have an occurrence of greater than 80% in the PB1 protein sequences from avian, human or mammalian IAVs. Moreover, the log-likelihood ratio tests were performed to test the dependence between the existence of a SLiM and the host origin of the PB1 protein. All 9 SLiMs have a *p* value of less than 0.05 indicate there are dependences between the existence of the 9 SLiMs and the host origin of PB1 proteins. As shown in Figure 3A, 8 of the 9 SLiMs have a lower occurrence in PB1 proteins from human IAVs than PB1 proteins from avian and mammalian IAVs. Notably, two of them (LIG_MAPK_1_584 and MOD_PAK_2_429) have a very low occurrence in PB1 proteins from human IAVs. In contrast, The SLiM MOD_PIKK_1_580 is specific to the PB1 proteins from human and mammalian IAVs. To reveal the underlying phylogenetic relationship, all PB1 sequences from each host class were used to perform pairwise alignments and the identities of all sequence pairs were computed (Figure 3B). Moreover, all sequences harbor a SLiM from each host class were used to perform pairwise alignments and the identities of all sequence pairs were computed. Two of the 9 SLiMs are shown in Figure 3C and 3D as examples. If two PB1 protein sequences with an identity greater than 95% are considered as sequences from the same lineage, then a SLiM identified from PB1 protein sequences with an identity greater than 95% may represent a result of founder effect. In contrast, a SLiM identified from PB1 protein sequences with an identity less than 95% may represent an event of host adaptation (convergent evolution). Results in Figure 3C and 3D suggest both of the founder effect and host adaptation were occurred. Similar phenomena were found for other SLiMs (Information S12).

### Comparison of the SLiM composition of the PB1 protein from IAVs of different levels of virulence

To uncover potential IAV virulence-associated motifs in PB1 proteins, a comparison of PB1 SLiM compositions from all IAVs and HP IAVs was conducted. The 126 SLiMs identified can be classified into three groups (Information S13). The first group is composed of 81 highly conserved SLiMs (with an occurrence of greater than 90% in all PB1 protein sequences) that are common in all PB1 proteins regardless of IAV virulence. The second group includes 12 partially conserved SLiMs (with an occurrence between 90–10% in all PB1 protein sequences). The third group has 33 low occurrence SLiMs (with an occurrence of less than 10% of all PB1 protein sequences). Therefore, the number of candidate motifs in the third group was reduced from 689 to 33. If a SLiM appears in PB1 proteins from HP IAVs but is very rare in PB1 proteins from human IAVs, it may be associated with the virulence of HP IAVs through its effect on the function of the PB1 protein. Using two criteria, a very low occurrence (less than 10%) in human IAV PB1 proteins and the presence in HP IAV PB1 proteins, 33 SLiMs from the second and third groups were identified. Moreover, two SLiMs from the second group were found that have a high occurrence in PB1 proteins from avian and mammalian IAVs but a low occurrence (approximately 20%) in PB1 proteins from human IAVs. The 35 SLiMs (Information S14) are candidate sites that might affect PB1 protein activity and might be associated with IAV transcription and/or replication efficiency. Notably, 2 of the 35 SLiMs (MOD_PAK_2_429 and LIG_MAPK_1_584) are both avian and/or mammalian IAV specific (labelled “A & M” in Information S14).

### Comparison of the SLiM compositions of PB2 proteins among IAVs from different hosts

A comparison of PB2 SLiM compositions among IAVs from avian (A_PB2), human (H_PB2) and mammalian (M_PB2) hosts reveals that the 712 identified SLiMs can be classified into three groups (Information S15). The first class is composed of 94 highly conserved SLiMs (with an occurrence of greater than 90% of all PB2 protein sequences) that are common in all PB2 proteins regardless of the IAV host range (Information S16). The 94 SLiMs may be basic motifs that are essential for normal PB2 protein functions. The second class includes 25 partially conserved SLiMs (with an occurrence between 90–10% of all PB2 protein sequences). The third class has 593 low occurrence SLiMs (with an occurrence of less than 10% of all PB2 protein sequences). In total, 23 locations that contain two or more overlapping SLiMs from the first group were found (red rectangles in Information S16).

**Figure 4:**
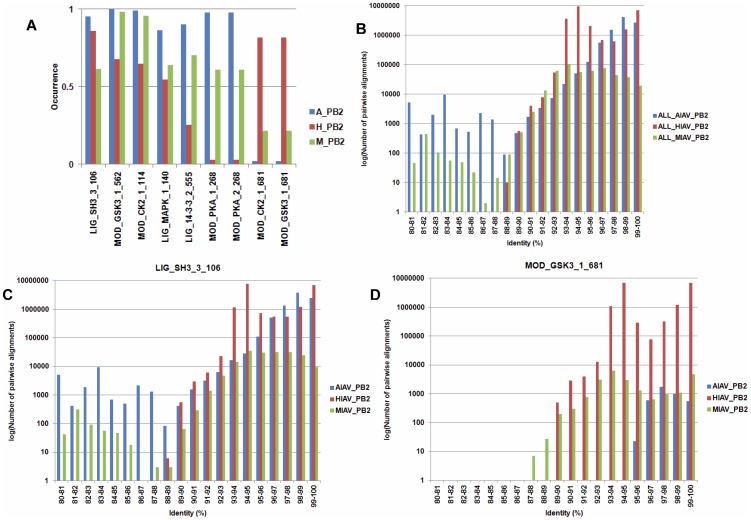
SLiMs from IAV PB2 proteins that have a differential occurrence in IAVs from different hosts. (A) 9 SLiMs that have a differential occurrence in IAV PB2 proteins from different hosts. A_PB2, H_PB2 and M_PB2 indicate the PB2 proteins from avian, human and IAV, respectively. The Y-axis indicates the occurrence of each identified SLiM. The X-axis indicates the name and position of each identified SLiM in the PB1 proteins. For example, “LIG_SH3_3” in “LIG_SH3_3_106” is the name of the SLiM, and 106 is the amino acid position where the SLiM starts. (B) The distribution of pairwise alignment identity of all PB2 protein sequences from avian, human and mammalian IAVs. (C) The distribution of pairwise alignment identity of PB2 protein sequences which harbor the SLiM LIG_SH3_3_106 from avian, human and mammalian IAVs. (D) The distribution of pairwise alignment identity of PB2 protein sequences which harbor the SLiM MOD_GSK3_1_681 from avian, human and mammalian IAVs. For B, C and D, the X-axis indicates the number of pairwise alignments of IAV PB2 protein sequences. The Y-axis indicates the identity of pairwise alignment (the percentage of identical amino acids that are the same in both PB2 sequences). Blue: PB2 protein sequences from avian IAVs. Red: PB2 protein sequences from human IAVs. Green: PB2 protein sequences from mammalian IAVs.

To uncover IAV host specific motifs in PB2 proteins in the second group, the test for difference among *k* proportions is performed. Using the *p* value of 10^−100^ as a cut-off value, 9 SLiMs that have an occurrence of greater than 80% in PB2 protein sequences from avian, human or mammalian IAVs were identified. Moreover, the log-likelihood ratio tests were performed to test the dependence between the existence of a SLiM and the host origin of the PB2 protein. All 9 SLiMs have a *p* value of less than 0.05 indicate there are dependences between the existence of the 9 SLiMs and the host origin of PB2 proteins. As shown in Figure 4A, 7 of the 9 SLiMs have lower occurrence in the PB2 proteins from human IAVs than the PB2 proteins from avian and mammalian IAVs. Notably, 3 of the 9 SLiMs (LIG_14-3-3_2_555, MOD_PAK_1_268 and MOD_PAK_2_268) have a very low occurrence in PB2 proteins from human IAVs. In contrast, 2 SLiMs (MOD_CK2_1_681 and MOD_GSK3_1_681) are specific to PB2 proteins from human and mammalian IAVs. To reveal the underlying phylogenetic relationship, all PB2 sequences from each host class were used to perform pairwise alignments and the identities of all sequence pairs were computed (Figure 4B). Moreover, all sequences harbor a SLiM from each host class were used to perform pairwise alignments and the identities of all sequence pairs were computed. Two of the 9 SLiMs are shown in Figure 4C and 4D as examples. If two PB2 protein sequences with an identity greater than 95% are considered as sequences from the same lineage, then a SLiM identified from PB2 protein sequences with an identity greater than 95% may represent a result of founder effect. In contrast, a SLiM identified from PB2 protein sequences with an identity less than 95% may represent an event of host adaptation (convergent evolution). Results in Figure 4C and 4D suggest both of the founder effect and host adaptation were occurred. Similar phenomena were found for other SLiMs (Information S17).

### Comparison of the SLiM composition of PB2 proteins from IAVs with different levels of virulence

To uncover potential IAV virulence-associated motifs in PB2 proteins, a comparison of PB2 SLiM compositions from all IAVs and HP IAVs was conducted. The 157 SLiMs identified can be classified into three groups (Information S18). The first group is composed of 94 highly conserved SLiMs (with an occurrence of greater than 90% in all PB2 protein sequences) that are common in all PB2 proteins regardless of IAV virulence. The second group includes 23 partially conserved SLiMs (with an occurrence between 90–10% in all PB2 protein sequences). The third group has 40 low occurrence SLiMs (with an occurrence less than 10% in all PB2 protein sequences). Therefore, the number of candidate motifs in the third group was reduced from 593 to 40. If a SLiM appears in the PB1 proteins from HP IAVs but is very rare in PB2 proteins from human IAVs, it may be associated with the virulence of HP IAVs through its effect on the function of the PB2 protein. Using two criteria, a very low occurrence (less than 10%) in human IAV PB2 proteins and the presence in HP IAV PB2 proteins, 41 SLiMs from the second and third groups were identified. Moreover, a SLiM from the second group was found that has a high occurrence in PB2 proteins from avian and mammalian IAVs but a low occurrence (25.4%) in PB2 proteins from human IAVs. The 42 SLiMs (Information S19) are candidates sites that might affect PB2 protein activity and might be associated with IAV transcription and/or replication efficiency. Importantly, 14 of the 42 SLiMs are even more notable. Three of them (MOD_CK2_1_336, LIG_FHA_2_337 and LIGTRAF2_1_339) are avian IAV specific (labelled “A” in Information S19). Another eight SLiMs (LIG_SH3_3_536, TRG_LysEnd_APsAcLL_1_441, MOD_PKA_2_659, LIG_APCC_KENbox_2_698, MOD_CK2_1_714, LIG_FHA_2_715, TRG_NLS_MonoCore_2_735 and TRG_NLS_MonoExtN_4_736) are mammalian IAV specific (labelled “M” in Information S19). Three SLiMs (MOD_PKA_1_268, MOD_PKA_2_268 and LIG_14-3-3_2_555) are avian and mammalian IAV specific (labelled “A & M” in Information S19).

### Comparison of the SLiM composition of NP proteins among IAVs from different hosts

A comparison of NP SLiM compositions among IAVs from avian (A_NP), human (H_NP) and mammalian (M_NP) hosts reveals that the 630 identified SLiMs can be classified into three groups (Information S20). The first class is composed of 37 highly conserved SLiMs (with an occurrence of greater than 90% in all NP protein sequences) that are common in all NP proteins regardless of IAV host range (Information S21). The 37 SLiMs may be basic motifs that are essential for normal NP protein functions. The second class includes 28 partially conserved SLiMs (with an occurrence between 90–10% in all NP protein sequences). The third class has 565 low occurrence SLiMs (with an occurrence of less than 10% in all NP protein sequences). 6 locations that contain two or more overlapping SLiMs from the first group were found (red rectangles in Information S21).

**Figure 5:**
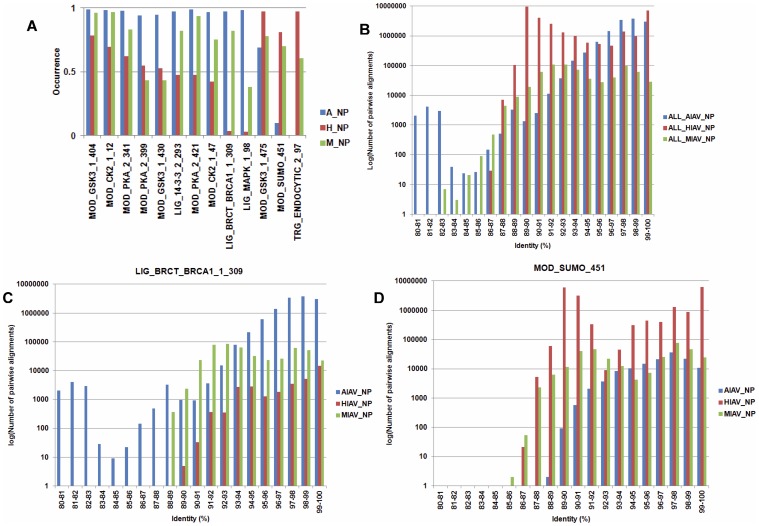
SLiMs from IAV NP proteins that have a differential occurrence in IAVs from different hosts. (A) 13 SLiMs that have a differential occurrence in IAV NP proteins from different hosts. A_NP, H_NP and M_NP indicate the NP proteins from avian, human and IAV, respectively. The Y-axis indicates the occurrence of each identified SLiM. The X-axis indicates the name and position of each identified SLiM in the NP proteins. For example, “MOD_SUMO” in “MOD_SUMO_451” is the name of the SLiM, and 451 is the amino acid position where the SLiM starts. (B) The distribution of pairwise alignment identity of all NP protein sequences from avian, human and mammalian IAVs. (C) The distribution of pairwise alignment identity of NP protein sequences which harbor the SLiM LIG_BRCT_BRCA1_1_309 from avian, human and mammalian IAVs. (D) The distribution of pairwise alignment identity of NP protein sequences which harbor the SLiM MOD_SUMO_451 from avian, human and mammalian IAVs. For B, C and D, the X-axis indicates the number of pairwise alignments of IAV NP protein sequences. The Y-axis indicates the identity of pairwise alignment (the percentage of identical amino acids that are the same in both NP sequences). Blue: NP protein sequences from avian IAVs. Red: NP protein sequences from human IAVs. Green: NP protein sequences from mammalian IAVs.

To uncover IAV host specific motifs in NP proteins in the second group, the test for differences among *k* proportions is performed. Using the *p* value of 10^−100^ as a cut-off value, 13 SLiMs that have an occurrence of greater than 80% in the NP protein sequences from avian, human or mammalian IAVs were identified. Moreover, the log-likelihood ratio tests were performed to test the dependence between the existence of a SLiM and the host origin of the NP protein. All 13 SLiMs have a *p* value of less than 0.05 indicate there are dependences between the existence of the 13 SLiMs and the host origin of NP proteins. As shown in Figure 5A, 10 of the 13 SLiMs have a lower occurrence in the NP proteins from human IAVs than in the NP proteins from avian and mammalian IAVs. Notably, 2 of them (LIG_BRCT_BRCA1_1_309 and LIG_MAPK_1_98) have a very low occurrence in NP proteins from human IAVs. In contrast, 2 SLiMs (MOD_SUMO_451 and TRG_ENDOCYTIC_2_97) are specific to the NP proteins from human and mammalian IAVs. To reveal the underlying phylogenetic relationship, all NP sequences from each host class were used to perform pairwise alignments and the identities of all sequence pairs were computed (Figure 5B). Moreover, all sequences harbor a SLiM from each host class were used to perform pairwise alignments and the identities of all sequence pairs were computed. Two of the 13 SLiMs are shown in Figure 5C and 5D as examples. If two NP protein sequences with an identity greater than 95% are considered as sequences from the same lineage, then a SLiM identified from NP protein sequences with an identity greater than 95% may represent a result of founder effect. In contrast, a SLiM identified from NP protein sequences with an identity less than 95% may represent an event of host adaptation (convergent evolution). Results in Figure 5C and 5D suggest both of the founder effect and host adaptation were occurred. Similar phenomena were found for other SLiMs (Information S22).

### Comparison of the SLiM composition of NP proteins from IAVs with different virulence

To uncover potential IAV virulence associated motifs in NP proteins, a comparison of NP SLiM compositions from all IAVs and HP IAVs was conducted. The 83 SLiMs identified can be classified into three groups (Information S23). The first group is composed of 37 highly conserved SLiMs (with an occurrence of greater than 90% in all NP protein sequences) that are common in all NP proteins regardless of IAV virulence. The second group includes 25 partially conserved SLiMs (with an occurrence between 90–10% in all NP protein sequences). The third group has 21 low occurrence SLiMs (with an occurrence of less than 10% in all NP protein sequences). Therefore, the number of candidate motifs in the third group was reduced from 565 to 21. If a SLiM appears in NP proteins from HP IAVs but is very rare in NP proteins from human IAVs, it may be associated with the virulence of the HP IAVs through its effect on the function of the NP protein. Using two criteria, a very low occurrence (less than 10%) in human IAV NP proteins and the presence in HP IAV NP proteins, 24 SLiMs from the second and third groups were identified. The 24 SLiMs (Information S24) are candidate sites that might affect NP protein activity and might be associated with IAV transcription and/or replication efficiency. In total, 6 of the 24 SLiMs are even more notable. 3 of them (LIG_APCC_KENbox_2_318, LIG_14-3-3_3_470 and MOD_ProDKin_1_470) are specific to the NP proteins from mammalian IAV (labelled “M” in Information S24). Another 3 SLiMs (LIG_MAPK_1_98, LIG_BRCT_BRCA1_1_309 and MOD_GSK3_1_370) are specific to the NP proteins from avian and mammalian IAV (labelled “A & M” in Information S24).

### SLiMs at the vicinity of amino acids that were associated with host adaptation of IAV PA proteins

Several amino acid sites (AASs) in IAV RNPs were reported to affect IAV RNP activity or were associated with IAV host adaptation [15]–[31]. In total, 99 AASs (25 in the PA protein, 16 in the PB1 protein, 31 in the PB2 protein and 27 in the NP protein) from these reports were mapped to 185 SLiMs (42 in the PA protein, 35 in the PB1 protein, 67 in the PB2 protein and 41 in the NP protein) identified in this study (Table 2–5). For instance, Gabriel et al used the highly pathogenic avian IAV SC35 to demonstrate that 7 AASs in IAV RNPs are associated with host adaptation [22]. All 7 of the AASs have corresponding SLiMs identified in this study. The AAS 615 of the PA protein can be mapped to LIG_FHA_2_612, LIG_14-3-3_3_615 and LIG_14-3-3_1_615 of the PA protein (Table 2). The AAS 13 of the PB1 protein can be mapped to MOD_PIKK_1_11 and MOD_GSK3_1_13 of the PB1 protein (Table 3). The AAS 678 of the PB1 protein can be mapped to MOD_PIKK_1_675 of the PB1 protein (Table 3). The AAS 333 of the PB2 protein can be mapped to MOD_PKA_1_331, MOD_PKA_2_331, MOD_CK2_1_335, MOD_CK2_1_336 and LIG_FHA_2_336 of the PB2 protein (Table 4). The AAS 701 of the PB2 protein can be mapped to LIG_MAPK_1_702 of the PB2 protein (Table 4). The AAS 714 of the PB2 protein can be mapped to MOD_CK2_1_714, LIG_FHA_2_715 and MOD_SUMO_717 of the PB2 protein (Table 4). The AAS 319 of the NP protein can be mapped to LIG_APCC_KENbox_2_318 and MOD_GSK3_1_319 of the NP protein (Table 5). Altogether, SLiMs identified in this study provide possible molecular mechanisms that may explain the activity, interaction or localization changes of IAV RNPs caused by those AAS changes.

**Table 2:** SLiMs mapped to the vicinity of amino acids that were reported as genetic signatures or are associated with the adaptation of IAV PA proteins to the host.

SLiMs identified in this study	Start	End	SLiM sequences	Amino acid	References
LIG_FHA_2_18	18	24	IVELA**EKTMKE**YGEDLK	20, 22	[23], [27]
MOD_SUMO_28	28	31	EYGED**PKIE**TNKFA	28	[19], [20], [25]
LIG_14-3-3_3_57	57	62	HFI**D**E**RSESII**VESGD	55, 57	[19], [20], [25]
LIG_14-3-3_2_57	57	63	HFI**D**E**RGESTIV**ESGDP	55, 57	[19], [20], [25]
MOD_GSK3_1_58	58	65	FI**D**E**RGESTIVES**GDPNA	55, 57	[19], [20], [25]
MOD_GSK3_1_62	62	69	RGESI**IVESGDPT**ALLKH	65, 66	[25], [26]
LIG_SH3_3_62	62	68	QGESI**IVEPEDP**NALLK	65, 66	[25], [26]
TRG_NES_CRM1_1_67	67	80	IVE**SGDLNALLKHRFEIIE**GRDRT	65, 66	[25], [26]
LIG_NRBOX_67	67	73	IVE**SGDLNALLK**HRFEI	65, 66	[25], [26]
MOD_GSK3_1_82	82	89	EIIEG**RDRTMAWT**VVNSI	85	[18]
LIG_PP1_82	82	89	EIIEG**RDRTIAWT**VINSI	85	[18]
LIG_FHA_2_96	96	102	VNSIC**NTTGVEK**PKFLP	97, 100	[19], [20], [25], [27], [28]
LIG_SH3_3_97	97	103	NSICN**TTGVEKP**KFLPD	97, 100	[19], [20], [25], [27], [28]
LIG_FHA_2_121	121	127	RFIEI**GVTRREV**HIYYL	127	[15]
LIG_SH2_STAT5_131	131	134	EVHIY**YLEK**ANKIK	133	[17]
MOD_GSK3_1_133	133	140	HIYYL**EKATKIKS**ENTHI	133	[17]
MOD_GSK3_1_187	187	194	MASR**GLWDSFRQS**ERGEE	186	[18]
LIG_SPAK-OSR1_1_204	204	208	ETIEE**RFEIT**GTMRK	204	[19]
MOD_CK2_1_221	221	227	DQSLP**PNFSSLE**NFRAY	225	[19], [20], [25]
LIG_CYCLIN_1_266	266	270	LKTTP**RPLRL**PNGPP	268	[19], [20], [25]
LIG_14-3-3_1_266	266	271	LKTTP**RPLTLP**DGPPC	268	[19], [20], [25]
LIG_FHA_2_267	267	273	KTTPR**PLTLPDG**PPCSQ	268	[19], [20], [25]
LIG_SH3_3_268	268	274	TTPRP**LRLPEGP**PCSQR	268	[19], [20], [25]
MOD_PIKK_1_274	274	280	RLPNG**PPCSQRS**KFLLM	277	[23]
LIG_WW_1_274	274	277	KLPNG**PPCY**QRSKF	277	[23]
LIG_SH3_3_319	319	325	FFGWK**EPTVVKP**HEKGI	321	[25]
LIG_SH2_STAT5_321	321	324	GWKEP**YIVK**PHEKG	321	[25]
LIG_SH2_STAT5_334	334	337	GINPN**YLLA**WKQVL	336	[15], [18]
LIG_CYCLIN_1_339	339	342	YLL**S**W**KQLL**AELQD	337	[20], [25]
LIG_Actin_WH2_2_353	353	369	FEDEK**KIPRVKNMKKTSPLKWA**LGENM	355, 356	[19], [23]
MOD_GSK3_1_356	356	363	EEKI**PRTKTMKKT**SQLKW	355, 356	[19], [23], [25], [29]
LIG_MAPK_1_358	358	366	KI**PK**T**KNMKKTIQL**KWALG	355, 356	[19], [23], [25], [29]
MOD_PKA_2_384	384	390	VDF**D**D**CRDTSDL**KQYDS	382	[25]
LIG_CYCLIN_1_401	401	405	DEPE**PRSLAI**WVQNE	400, 404	[19], [20], [25]
MOD_GSK3_1_402	402	409	EPE**L**R**SLASWIQS**EFNKA	400, 404, 409	[19], [20], [25]
MOD_CK2_1_418	418	424	KACEL**TDSSWIE**LDEIG	421	[25]
LIG_FHA_2_419	419	425	ACELT**DSTWIEL**DEIGE	421	[25]
LIG_SH3_3_554	554	560	LLR**T**A**VGQVSRP**MFLYV	552, 556	[17], [19], [20], [25], [29]
LIG_FHA_2_612	612	618	MTKEF**FETKSET**WPIGE	615	[15], [22]
LIG_14-3-3_3_615	615	620	EFFET**KSETWP**IGESP	615	[15], [22]
LIG_14-3-3_1_615	615	620	EFFEN**RSETWP**IGESP	615	[15], [22]

**Table 3 pone-0038637-t003:** SLiMs mapped to the vicinity of amino acids that were reported as genetic signatures or are associated with the adaptation of IAV PB1 proteins to the host.

SLiMs identified in this study	Start	End	SLiM sequences	Amino acid	References
MOD_PIKK_1_11	11	17	TLLF**LKVPTQNA**ISTTF	13	[22], [27]
MOD_GSK3_1_13	13	20	LFLKV**PAQSAIST**TFPYT	13	[22]
LIG_14-3-3_2_52	52	58	HQYSE**RGKWTIN**TETGA	52	[29]
MOD_PKA_2_53	53	59	QYSE**RGRWTTNT**ETGAP	52	[29]
MOD_CK2_1_54	54	60	YSE**K**G**KWTTNTE**TGAPQ	52	[29]
MOD_GSK3_1_54	54	61	YSE**K**G**KWTTNTET**GAPQL	52	[29]
MOD_CK2_1_103	103	109	SHPGI**FENSCLE**TMEVV	103	[23]
MOD_GSK3_1_103	103	110	SHPGI**FENSCIET**MEVVQ	103	[23]
TRG_LysEnd_APsAcLL_1_214	214	219	KQKLN**RKGYLI**RALTL	216	[19]
LIG_SH2_STAT5_217	217	220	VNKR**GYLIR**ALTLN	216	[19]
MOD_PIKK_1_290	290	296	VVRKM**MTNSQDT**ELSFT	292	[23]
LIG_APCC_Dbox_1_315	315	323	NENQN**PRMFLAMIT**YITKN	317	[15]
LIG_14-3-3_2_316	316	322	ENQNP**RVFLTMI**TYITK	317	[15]
LIG_SH2_STAT5_324	324	327	LAMIT**YITR**NQPEW	327	[19]
MOD_PIKK_1_325	325	331	AMITY**ITRSQPE**WFRNV	327	[19]
MOD_CK2_1_325	325	331	AMITY**ITRSQPE**WFRNV	327	[19]
LIG_14-3-3_2_334	334	340	QPEWF**RNILSIA**PIMFS	336	[19], [25]
LIG_FHA_2_372	372	378	QIPAE**MLTSIDL**KYFNE	372	[23]
LIG_SH2_GRB2_380	380	383	SIDLK**YFNES**TRKK	384	[15]
LIG_SH2_STAT5_380	380	383	SIDLK**YFNES**TRKK	384	[15]
MOD_CK2_1_384	384	390	KYFNE**STRTKIE**KIRPL	384	[15]
LIG_FHA_2_385	385	391	YFNE**STRTKIEK**IRPLL	384	[15]
LIG_MAPK_1_386	386	397	FNE**S**T**RKKIEKIRPLLI**DGTAS	384	[15]
MOD_GSK3_1_517	517	524	FGVSG**INESADMSI**GVTV	517	[29]
LIG_PTB_Apo_2_551	551	558	MALQL**FIKNYRYT**YRCHR	552	[15]
LIG_PTB_Phospho_1_551	551	557	MALQL**FIKNYRY**TYRCH	552	[15]
MOD_PKA_2_555	555	561	LF**I**KD**YRYTYRC**HRGDT	552	[15]
MOD_CK2_1_575	575	581	TRRSF**ELKTLWE**QTRSK	578	[26]
LIG_FHA_2_576	576	582	RRSFE**LKTLWDQ**TQSKA	578	[26]
MOD_PIKK_1_580	580	586	ELKKL**WDQTQSK**AGLLV	584	[29]
MOD_GSK3_1_580	580	587	ELKKL**WDQTQSRT**GLLVS	584	[29]
LIG_MAPK_1_584	584	591	LWEQT**RSKAGLLV**SDGGP	584	[29]
MOD_SUMO_611	611	614	IPEVC**LKWE**LMDED	614	[26]
LIG_Clathr_ClatBox_1_615	615	619	CLKW**ELMDVD**YQGRL	614	[26]
MOD_PIKK_1_675	675	681	RNRSI**LNTSQRG**ILEDE	677, 678	[16], [22]

**Table 4 pone-0038637-t004:** SLiMs mapped to the vicinity of amino acids that were reported as genetic signatures or are associated with the adaptation of IAV PB2 proteins to the host.

SLiMs identified in this study	Start	End	SLiM sequences	Amino acid	References
LIG_CYCLIN_1_8	8	11	RIKEL**RDLM**SQSRT	9	[25]
MOD_GSK3_1_9	9	16	IKELR**DLMSQSRT**REILT	9	[25]
MOD_PIKK_1_9	9	15	IKELR**DLMSQSR**TREIL	9	[25]
LIG_PP1_44	44	50	QEKNP**SLRVKWM**MAMKY	44	[19], [20], [25], [29]
MOD_CK2_1_63	63	69	TADKR**IIETIPER**NEQG	63, 64, 70	[16], [20], [25], [27], [31]
LIG_FHA_2_64	64	70	ADKR**IIETIPER**NEQGQ	63, 64, 70	[16], [20], [25], [27], [31]
MOD_GSK3_1_79	79	86	GQTLW**SKMSDAGS**DRVMV	81	[25]
MOD_PKA_2_79	79	85	GQTLW**SRMSDAG**SDRVM	81	[25]
MOD_PKA_2_104	104	110	WNRNG**PRTSAVH**YPKVY	105	[23], [25], [29]
LIG_SH3_3_106	106	112	RNGP**MTSTVHYP**KIYKT	105, 108	[23], [29]
MOD_CK2_1_152	152	158	INPGH**ADLSAKE**AQDVI	153, 156	[23], [31]
LIG_SH2_STAT5_195	195	198	KEELQ**YCKIA**PLMVA	199	[19], [20], [25], [29]
TRG_ENDOCYTIC_2_195	195	198	KEELQ**YCKIA**PLMVA	199	[19], [20], [25], [29]
MOD_ProDKin_1_196	196	202	EELQD**CKISPLM**VAYML	199	[19], [20], [25], [29]
MOD_PKA_1_268	268	274	ARNIV**RRATVSA**DPLAS	271	[19], [20], [21], [29], [31]
MOD_PKA_2_268	268	274	ARNIV**RRATVSA**DPLAS	271	[19], [20], [21], [29], [31]
MOD_GSK3_1_317	317	324	KAAMG**LRISSSFS**FGGFT	318	[15]
MOD_PKA_2_317	317	323	KAAMG**LRISSSF**SFGGF	318	[15]
LIG_14-3-3_2_318	318	324	AAMGL**RISSSLS**FGGFT	318	[15]
LIG_BRCT_BRCA1_1_319	319	323	AMGL**RISSSF**SFGGF	318	[15]
MOD_PKA_1_331	331	337	GGFTF**KRTSGSS**V**K**REE	333, 339	[22], [31]
MOD_PKA_2_331	331	337	GGFTF**KRTSGSS**V**K**REE	333, 339	[22], [31]
MOD_CK2_1_335	335	341	FKR**T**S**GSSVKRE**EEVLT	333, 339, 341	[22], [31]
MOD_CK2_1_336	336	342	KR**T**SG**SSVTKEE**EVLTG	333, 339, 341	[22], [31]
LIG_FHA_2_336	336	342	KR**T**SG**SSTKREE**EVLTG	333, 339, 341	[22], [31]
MOD_CK2_1_352	352	358	GNLQT**LKLTVHE**GYEEF	355	[15]
LIG_FHA_2_353	353	359	NLQTL**KLTVHEG**YEEFT	355	[15]
MOD_PKA_2_368	368	374	FTMVG**RRATAIL**RKATR	368	[25]
MOD_PKA_1_368	368	374	FTMVG**RRATAIL**RKATR	368	[25]
LIG_MAPK_1_476	476	485	TEMS**MRGIRVSKMGV**DEYSS	475	[19], [20], [25], [29]
LIG_CYCLIN_1_476	476	480	TEMS**MRGLRV**SKMGV	475	[19], [20], [25], [29]
LIG_FHA_2_481	481	487	RGVRV**SKTGVDE**YSSTE	482	[31]
TRG_NES_CRM1_1_492	492	506	EYSST**ERIVVSIDRFLRVRD**QRGNV	493	[29]
LIG_14-3-3_2_493	493	499	YSSTE**RVVVSID**RFLRV	493	[29]
MOD_ProDKin_1_530	530	536	ERLT**ITYSSPMM**WEING	529, 530	[15]
LIG_PP1_559	559	565	IRNWE**AVKIQWS**Q**N**PAM	567	[19], [20], [25], [31]
MOD_GSK3_1_562	562	569	WETVK**IQWSQDPTM**LYNK	567, 569, 570	[19], [20], [25], [29], [31]
MOD_PIKK_1_562	562	568	WETVK**IQWSQDP**T**M**LYN	567, 569, 570	[19], [20], [25], [29], [31]
MOD_PIKK_1_587	587	593	SLVPK**AIRSQYS**GFVRT	588, 591	[19], [20], [21], [24], [25]
MOD_PKA_2_590	590	596	PKA**T**R**SRYSGFV**RTLFQ	588, 591	[19], [20], [21], [24], [25]
MOD_PIKK_1_610	610	616	DVLGT**FDTTQII**KLLPF	611, 613	[19], [25], [29], [31]
MOD_GSK3_1_659	659	666	SPVFNY**NKATKRLT**VLGKD	661	[25], [29]
MOD_PIKK_1_659	659	665	PVFNY**NKATQRL**TVLGK	661	[25], [29]
MOD_PKA_2_659	659	665	PVFNY**NRATKRL**TVLGK	661	[25], [29]
LIG_CYCLIN_1_663	663	667	YNK**A**T**KRLTV**LGKDA	661	[25], [29]
MOD_PKA_1_663	663	669	YNK**A**T**KRLTVLG**KDAGA	661	[25], [29]
MOD_PKA_2_663	663	669	YNK**A**T**KRLTVLG**KDAGA	661	[25], [29]
MOD_CK2_1_671	671	677	TILGK**DAGTLIE**DPDES	674	[20], [25]
TRG_LysEnd_APsAcLL_1_671	671	676	TILGK**DAGTLI**EDPDE	674	[20], [25]
LIG_FHA_2_672	672	678	VLGKD**AGTLTED**PDEGT	674	[20], [25]
MOD_CK2_1_681	681	687	IEDP**DESTSGVE**SAVLR	680, 682, 684	[19], [29], [31]
MOD_GSK3_1_681	681	688	TEDP**DEGTSGVES**AVLRG	680, 682, 684	[19], [29], [31]
LIG_FHA_2_682	682	688	EDP**D**E**GTTGVES**AVLRE	680 682, 684	[19], [29], [31]
LIG_APCC_KENbox_2_698	698	702	GFLIL**GKENK**RYGPA	698, 701, 702	[15], [17], [19], [20], [21], [22], [23], [25], [29], [30], [31]
LIG_MAPK_1_702	702	710	LGKE**DKRYGPALSI**NELSN	701, 702	[15], [17], [19], [20], [21], [22], [23], [25], [29], [30], [31]
MOD_CK2_1_714	714	720	SINEL**SNLTKGE**KANVL	714	[22]
LIG_FHA_2_715	715	721	INEL**SNLTKGEK**ANVLI	714	[22]
MOD_SUMO_717	717	720	EL**S**NL**AKGE**KANVL	714	[22]
MOD_GSK3_1_738	738	745	LVMKR**KRDSSILT**DSQTA	740	[29]
MOD_PKA_2_738	738	744	LVMKR**KRDSSIL**TDSQTA	740	[29]
MOD_PKA_1_738	738	744	LVMKR**KRDSSIL**TDSQT	740	[29]
MOD_GSK3_1_742	742	749	RKR**D**S**SILTDSQT**ATKRI	740	[29]

**Table 5:** SLiMs mapped to the vicinity of amino acids that were reported as genetic signatures or are associated with the adaptation of IAV NP proteins to the host.

SLiMs identified in this study	Start	End	SLiM sequences	Amino acid	References
TRG_ENDOCYTIC_2_10	10	13	GTKR**SYEQM**ETGGE	9	[23]
MOD_CK2_1_12	12	18	KR**S**YE**QMETGGE**RQDAT	9, 16	[19], [20], [23], [25]
MOD_PKA_2_18	18	24	MET**D**G**ERQTATE**IRASV	16	[19], [20], [25]
LIG_SH2_STAT5_78	78	81	ERRN**KYLEE**HPSAG	77	[29]
LIG_MAPK_1_98	98	110	GGPIY**RRRDGKWMRELIL**YDKEE	100	[19], [25]
LIG_Actin_WH2_2_101	101	119	IYRR**VDGKWMRELILYDKEELRRV**WRQAN	100, 101, 102	[19], [25], [29]
LIG_MAPK_1_102	102	110	YKR**VDRKWMRELVL**YDKEE	100, 101, 102	[19], [25], [29]
LIG_CYCLIN_1_106	106	110	DGKWM**RELIL**YDKEE	109	[19]
MOD_GSK3_1_127	127	134	QANNG**EDATAGLT**HIMIW	131	[29]
MOD_PKA_2_212	212	218	WRGEN**GRRTRIA**YERMC	214	[19], [25]
MOD_CK2_1_214	214	220	GENGR**KTRSAYE**RMCNI	214	[19], [25]
MOD_CK2_1_284	284	290	RESR**NPGNTEIE**DLIFL	283	[19], [20], [25], [29]
LIG_14-3-3_2_292	292	298	GHDF**EREGYSLV**GIDPF	291, 293	[19], [23]
LIG_AP2alpha_2_302	302	304	SLVGI**DPFK**LLQN	305	[19], [20], [25], [29]
MOD_PIKK_1_307	307	313	DPF**R**L**LQNSQVF**SLIRS	305	[19], [20], [25], [29]
MOD_GSK3_1_307	307	314	DPF**R**L**LQNSQVFS**LIRSN	305	[19], [20], [25], [29]
TRG_ENDOCYTIC_2_313	313	316	QNSQV**YSLI**RPNEN	313	[19], [20], [25]
LIG_APCC_KENbox_2_318	318	322	YSLIR**PKENP**AHKSQ	319	[22]
MOD_GSK3_1_319	319	326	SLIRP**KENSAHKS**QLVWM	319	[22]
LIG_BRCT_BRCA1_1_334	334	338	VWMAC**HSAAF**EDLRV	335	[29]
LIG_14-3-3_3_334	334	339	VWMAC**HSASFE**DLRVS	335	[29]
LIG_FHA_2_335	335	341	WMACH**SATFEDL**RVSSF	335	[29]
MOD_ProDKin_1_350	350	356	SFIRG**TKVSPRG**KLSTR	353	[23], [29]
LIG_CYCLIN_1_351	351	354	FIRGT**RVLP**RGKLS	353	[23], [29]
MOD_PKA_2_356	356	362	KVSPR**GRLSTRG**VQIAS	357	[19], [20], [25], [29]
LIG_Actin_WH2_2_367	367	384	GVQ**I**A**SNENMDNMGSGTLELRSG**YWAIR	365	[23]
MOD_GSK3_1_370	370	377	IASNE**NMETMDSS**TLELR	372, 375, 377	[19], [23], [25], [29]
MOD_CK2_1_374	374	380	ENM**D**N**MGSSTLE**LRSGY	372, 375, 377	[19], [23], [25], [29]
LIG_FHA_2_375	375	381	NVEAM**DSTTLEL**RSRYW	375, 377	[23], [25], [29]
MOD_PKA_2_421	421	427	RSLPF**ERATIMA**AFTGN	422, 423, 425	[19], [25], [29]
MOD_GSK3_1_450	450	457	IRMME**GAKTEEVS**FRGRG	450, 455	[19], [23]
LIG_TRAF6_451	451	459	RMME**GAKPEEVSFQ**GRGVF	450, 455	[19], [23]
MOD_SUMO_451	451	454	RMME**GAKPEE**VSFQ	450, 455	[19], [23]
LIG_CtBP_453	453	457	MESAK**PEDLS**FQGRG	455	[19]
LIG_14-3-3_3_470	470	475	ELSDE**KATSPI**VPSFD	472	[29]
MOD_ProDKin_1_470	470	476	ELSDE**KATSPIV**PSFDM	472	[29]
LIG_SH3_3_471	471	477	LSDEK**ATNPIVP**SFDMS	472	[29]

### Proposed cellular processes RNPs may be involved through SLiMs identified

The compositions of SLiMs in RNPs provide information regarding the pathways that the RNPs may be involved in. As shown in Table 6, RNPs with SH2 and SH3 ligand motifs, LIG_MAPK_1, LIG_14-3-3, LIG_FHA_2 and protein kinase phosphorylation sites may be involved in the MAPK, Wnt and PI3K/AKT/FOXO signal transduction pathways. RNPs with LIG_TRAF2_1, LIG_TRAF6, LIG_SH2_STAT3 and LIG_SH2_STAT5 may be involved in the TNF/cytokine signaling pathway [3]. Moreover, RNPs with TRG_LysEnd_APsAcLL_1, TRG_ENDOCYTIC_2, LIG_EH1_1 and LIG_Actin_WH2_2 may interact with actin and be involved in intracellular trafficking pathways [3]. All of these host cellular processes and pathways have been reported to be involved in post-entry steps of IAV replication [32], [33]. The different compositions of SLiMs among RNPs reflect the functional diversity of RNPs. Each RNP with a different SLiM composition has a varying ability to interact with different cellular processes and signal transduction pathways, and results in different impacts on viral replication and host adaptation.

**Table 6:** Functions of SLiMs identified in IAV RNPs. In the ELM database [3], SLiMs are divided into four types: protease cleavage sites (prefix CLV), protein motif interacting/binding sites (prefix LIG), posttranslational modification sites (prefix MOD) and subcellular targeting signals (prefix TRG).

ELM Identifier	Description	GO_ID	GO_Process	RNPs
CLV_PCSK_FUR_1	The furin cleavage site	GO:0006508	Proteolysis And Peptidolysis	PB1
LIG_14-3-3_1	The 14-3-3 protein interacting motif	GO:0007243	Protein Kinase Cascade	PA, PB2
LIG_14-3-3_2	The 14-3-3 protein interacting motif	GO:0007243	Protein Kinase Cascade	PA, PB1, PB2, NP
LIG_14-3-3_3	The 14-3-3 protein interacting motif	GO:0007243	Protein Kinase Cascade	PA, PB1, PB2, NP
LIG_Actin_WH2_2	The actin interacting motif	GO:0008064	Regulation Of Actin Polymerization Or Depolymerization	PA, PB2, NP
LIG_AGCK_PIF_1	The PIF motif interacting motif	GO:0007165	Signal Transduction	PB2
LIG_AP2alpha_2	The AP2 complex interacting motif	GO:0006897	Endocytosis	PA, NP
LIG_APCC_Dbox_1	The APC/C complex interacting motif	GO:0043161	Proteasomal Ubiquitin-Dependent Protein Catabolic Process	PA, PB1, PB2
LIG_APCC_KENbox_2	The APC/C complex interacting motif	GO:0043161	Proteasomal Ubiquitin-Dependent Protein Catabolic Process	PA, PB2, NP
LIG_BRCT_BRCA1_1	The BRCT domain interacting motif	GO:0000077	Dna Damage Checkpoint	PA, PB1, PB2, NP
LIG_Clathr_ClatBox_1	The N-terminus of Clathrin heavy chain interacting motif	GO:0006897	Endocytosis	NP
LIG_CtBP	The CtBP protein interacting motif	GO:0045449	Regulation Of Transcription	PA, NP
LIG_CYCLIN_1	Cyclin interacting motif	GO:0007049	Cell Cycle	PA, PB1, PB2, NP
LIG_EH_1	EH domain interacting motif	GO:0006897	Endocytosis	PB1
LIG_FHA_2	FHA domain interacting motif	GO:0045449	Regulation Of Transcription	PA, PB1, PB2, NP
LIG_HCF-1_HBM_1	the Host Cell Factor-1 interacting motif	GO:0045449	Regulation Of Transcription	PB2
LIG_HP1_1	HP1 protein interacting motif	GO:0006343	Establishment Of Heterochromatin Silencing	PA, PB2
LIG_MAD2	Mad2 interacting motif	GO:0007094	Mitotic Spindle Checkpoint	PB2
LIG_MAPK_1	MAPK interacting motif	GO:0007243	Protein Kinase Cascade	PA, PB1, PB2, NP
LIG_NRBOX	Nuclear receptor interacting motif	GO:0045449	Regulation of Transcription	PA
LIG_PDZ_Class_2	The C-terminal class 2 PDZ-binding motif	GO:0007165	Signal Transduction	NP
LIG_PP1	The Protein phosphatase 1 catalytic subunit interacting motif	GO:0007165	Signal Transduction	PA, PB1, PB2
LIG_PTB_Apo_2	The Dab-like PTB domain interacting motif	GO:0007169	Transmembrane Receptor Protein Tyrosine Kinase Signalimg Pathway	PA, PB1
LIG_PTB_Phospho_1	The Shc-like and IRS-like PTB domain interacting motif	GO:0007169	Transmembrane Receptor Protein Tyrosine Kinase Signalimg Pathway	PA, PB1
LIG_RGD	The RGD motif binding motif	GO:0007155	Cell-Adhesion	PB1, PB2, NP
LIG_SH2_GRB2	The GRB2-like Src Homology 2 (SH2) domain binding motif	GO:0007165	Signal Transduction	PA, PB1, PB2
LIG_SH2_SRC	The Src-family Src Homology 2 (SH2) domains binding motif	GO:0007165	Signal Transduction	PA, PB2
LIG_SH2_STAT3	The STAT3 SH2 domain interacting motif	GO:0045449	Regulation Of Transcription	PB1
LIG_SH2_STAT5	The STAT5 SH2 domain binding motif	GO:0045449	Regulation Of Transcription	PA, PB1, PB2, NP
LIG_SH3_3	The SH3 domain interacting motif	GO:0007165	Signal Transduction	PA, PB1, PB2, NP
LIG_SPAK-OSR1_1	SPAK/OSR1 kinase binding motif	GO:0006468	Protein Amino Acid Phosphorylation	PA, NP
LIG_SxIP_EBH_1	The EBH domain interacting motif	GO:0031535	Plus-End Directed Microtubule Sliding	PB1, NP
LIG_TRAF2_1	TRAF2 binding motif	GO:0007165	Signal Transduction	PA, PB1, PB2, NP
LIG_TRAF6	TRAF6 binding motif	GO:0007165	Signal Transduction	PB1, PB2, NP
MOD_CDK_1	CDK phosphorylation site	GO:0000308	Cycloplasmic Cyclin-Dependent Protein Kinase Holoenzyme Complex	PA
MOD_CK2_1	CK2 phosphorylation site	GO:0016055	Wnt Receptor Signaling Pathway	PA, PB1, PB2, NP
MOD_GSK3_1	GSK3 phosphorylation site	GO:0006468	Protein Amino Acid Phosphorylation	PA, PB1, PB2, NP
MOD_PIKK_1	PIKK phosphorylation site	GO:0000077	Dna Damage Checkpoint	PA, PB1, PB2, NP
MOD_PKA_1	PKA-type AGC kinase phosphorylation site	GO:0010737	Protein Kinase A Signaling Cascade	PA, PB1, PB2
MOD_PKA_2	PKA-type AGC kinase phosphorylation site	GO:0010737	Protein Kinase A Signaling Cascade	PA, PB1, PB2, NP
MOD_ProDKin_1	Proline-Directed Kinase phosphorylation site	GO:0007165	Signal Transduction	PA, PB1, PB2, NP
MOD_SUMO	Motif for modification by SUMO-1	GO:0016927	Sumoylation	PA, PB1, PB2, NP
MOD_TYR_ITSM	immunoreceptor tyrosine based switch motif	GO:0006468	Protein Amino Acid Phosphorylation	PB2
TRG_ENDOCYTIC_2	Tyrosine-based sorting signal interacts with adaptor complexes	GO:0006897	Endocytosis	PA, PB1, PB2, NP
TRG_LysEnd_APsAcLL_1	Sorting and internalisation signal interacts with adaptor complexes	GO:0006886	Intracellular Protein Transport	PA, PB1, PB2, NP
TRG_NES_CRM1_1	Nuclear export signal (NES) binding to the CRM1 exportin protein	GO:0051168	Nuclear Export	PA, PB2, NP
TRG_NLS_Bipartite_1	Bipartite nuclear localization signal	GO:0006606	Protein-Nucleus Import	PB1, PB2, NP
TRG_NLS_MonoCore_2	Monopartite nuclear localization signal, Strong core type	GO:0006607	Nls-Bearing Substrate Import Into Nucleus	PA, PB1, PB2
TRG_NLS_MonoExtC_3	Monopartite nuclear localization signal, C-extended type	GO:0006607	Nls-Bearing Substrate Import Into Nucleus	PB1
TRG_NLS_MonoExtN_4	Monopartite nuclear localization signal, N-extended type	GO:0006607	Nls-Bearing Substrate Import Into Nucleus	PB1, PB2

## Discussion

In total, 292 highly conserved SLiMs were found in IAV RNPs regardless of IAV host range. These SLiMs may be basic motifs that are essential for the normal function of RNPs. Two of them have been experimentally identified in IAV RNP proteins. The first SLiM is the nuclear localization signal (NLS) located between amino acid 182–217 in the IAV PB1 proteins [34]. Several NLS associated SLiMs were identified in this study as shown in Information S11. The second SLiM is the nuclear localization signal (NLS) located in the C-terminal of the IAV PB2 proteins [35]. The NLS associated SLiM was identified in this study as shown in Information 16. These examples suggest that computational prediction of SLiM is helpful for identification of important function motifs in viral proteins.

In total, 67 locations with overlapping SLiMs were identified among the 292 highly conserved SLiMs in RNPs (red rectangles in Information S6, S11, S16, S21). These overlapping SLiMs may act together through three mechanisms. First, multiple SLiM interactions may be used cooperatively to increase the specificity and strength with which two proteins bind to each other. Second, multiple SLiMs may enable interaction between different cellular signals sequentially. For example, the function of the first SLiM may lead to the action of the second SLiM. Third, multiple SLiMs may also enable the interaction between different cellular signals competitively. A protein may contain different SLiMs that target the same amino acid residue for different post translational modifications as inputs from different cellular signals. This could lead to competition (i.e. an interaction) between the two signals, with different enzymes competing to modify the same residue. The different post translational modification states of the motif could bind to different interaction domains and result in different output signals from the interaction.

The SLiMs which have a very low occurrence in RNPs from human IAVs but present in RNPs from HP IAVs could be candidates for novel virulent determinants that are worthy to be further investigated. For example, 10 SLiMs (LIG_SPAK-OSR1_1_204, MOD_PIKK_1_274 and MOD_GSK3_1_402 in PA proteins; MOD_PAK_2_429 and LIG_MAPK_1_584 in PB1 proteins; MOD_PKA_1_268, MOD_PKA_2_268 and LIG_14-3-3_2_555 in PB2 proteins; and LIG_MAPK_1_98 and LIG_BRCT_BRCA1_1_309 in NP proteins) have a very low occurrence in RNPs from human IAVs but have a high occurrence in RNPs from avian and mammalian IAVs. Moreover, all 10 of the SLiMs were found in RNPs from HP IAVs (Information S9, S14, S19, S24). Therefore, they may represent emerging SLiMs in RNPs of avian IAV origin which are in the early stage of adaptation to human hosts. Another type of SLiMs that have a low occurrence in RNPs from avian, human and mammalian IAVs but are present in HP IAVs may also be potential virulent determinants that occurred by coincidence (Information S9, S14, S19, S24).

Many proteins are regulated by post-translational modifications (PTMs) that may mediate allosteric effects or create binding sites important for protein-protein interactions where ligand domains can bind to phosphorylated, methylated or sumoylated sites. As described for the ELM server, SLiMs can be classified into four types of functional sites: ligand sites (LIG), PTM sites (MOD), proteolytic cleavage and processing sites (CLV), and sites for subcellular targeting (TRG) [3]. These functional assignments are useful in that they encompass the range of peptide motif activities. Furthermore, they can also help explain why many amino acid sites have been experimentally demonstrated to be functionally important for RNPs but do not have corresponding SLiMs in this study. For example, the glutamic acid at PB2 position 627 is generally found in avian viruses, whereas nearly all human isolates carry a lysine at this position [36]. Available data suggests that PB2 position 627 determines the temperature sensitivity of vRNA replication [37]. Viruses with PB2 627K can efficiently replicate in the mammalian upper respiratory tract, whereas those that possess PB2 627E cannot [38]. A PB2 E627K mutation enhances avian virus replication in mammalian cells at 33°C, but not at 37°C or 41°C, in vitro [37]. A lack of a corresponding SLiM suggests the cold sensitivity of avian virus polymerases with PB2 627E may be because the global domain conformation changes in the PB2 protein are directly affected by the residue itself rather than mediated by a gain or loss of a post-translational modification target site (SLiM).

To validate the putative SLiMs identified in this study several experimental methods can be used. The first method is the reverse genetics technology that is generally used for validation of IAV protein function/activity affected by different amino acid mutations [39], [40]. The reverse genetics can be coupled with different function assays. For example, to validate the influence of a SLiM in virulence, virus particles produced by reverse genetics can be used to infect model animals (mouse, ferret, swine or primate). The survival rate, pathological changes, cytokine levels in blood could be measured [41], [42]. Interactions between IAV RNPs and known host factors through SLiMs (e.g. LIG_SH2_STAT5 and LIG_TRAF2) identified in this study can be validated by biomolecular fluorescence complementation (BiFC) [43], [44] and split luciferase complementation assay (SLCA) [45]. Localization of RNPs mediated by targeting signal SLiMs such as nuclear export signal and nuclear localization signal can be validated by fluorescence recovery after photobleaching (FRAP) [46]. Specific modification such as sumoylation can be validated by immunoblot of SUMO specific antibody [47], [48].

Using protein-protein interactions as targets for antiviral chemotherapy has been proposed over a decade [49]. Currently, this idea is considered in development of antiviral drugs for flaviviruses and HIV [50], [51]. To interfere in protein-protein interactions, using peptides that mimic the interaction motifs is one of the most straightforward approaches [52]. Several reports demonstrated that peptide-mediated interference in IAV polymerase complex assembly can attenuate IAV replication [53]–[57]. SLiMs such as PDZ motif [58], LIG_SH2_GRB2 [59] are being explored as drug targets. Since viruses have evolved to use motifs for essential functions by hijacking host proteins [60], identification of SLiMs which mediate interactions between viral protein and host factors may provide valuable and specific information for development of motif mimetic drugs to perturb the interactions to treat virus infections [2].

Inhibition of interactions between viral proteins has the advantage of high specificity and low side effect. However, resistant strains may appear from fast co-evolution of RNA virus proteins under selection pressures. The possibility of co-evolution of RNA virus proteins and mammalian host proteins, on the other hand, is expected to be extremely low. Another concern is that the inability of a synthetic peptide to penetrate cells precluded it from therapeutic usefulness. Nevertheless, discovery of peptidomimetic compounds can be pursued based on the structure of the effective peptide.

In this study, the compositions of SLiMs (target sites of post-translational modifications) of IAV RNPs were analyzed. Three groups of SLiMs with different occurrences for each RNP were found. The SLiMs identified in this study provide an invaluable resource for experimental virologists to study the interactions between IAV RNPs and host intracellular proteins. Moreover, the SLiM compositions of IAV RNPs also provide insights into the signal transduction pathways and protein interaction networks with which IAV RNPs might be involved or interfere. The information of SLiM mediated virus-host protein interactions might be helpful for the development of anti-IAV drugs.

## Supporting Information

Information S1 **Number of IAV ribonucleoprotein sequences used in this study.**
(DOC)

Information S2 **Lists of avian and mammalian hosts of IAV. Numbers of IAV ribonucleoprotein sequences from each host are included.**
(XLS)

Information S3 **Number of ribonucleoprotein sequences from highly virulent/pandemic IAVs used in this study.**
(DOC)

Information S4 **Information of 96 SLiMs used in this study.**
(TXT)

Information S5 **Detail information of comparisons of PA protein SLiM compositions among IAVs from different hosts.**
(XLS)

Information S6 **Highly conserved SLiMs in IAV PA proteins.**
(DOC)

Information S7 **The identity distributions of SLiMs from IAV PA proteins that have differential occurrences in IAVs from different hosts.**
(DOC)

Information S8 **Detail information of comparisons of PA protein SLiM compositions from IAVs of different virulence.**
(XLS)

Information S9 **SLiMs that are not highly conserved but appear in virulent/pandemic IAV PA proteins.**
(DOC)

Information S10 **Detail information of comparisons of PB1 protein SLiM compositions among IAVs from different hosts.**
(XLS)

Information S11 **Highly conserved SLiMs in IAV PB1 proteins.**
(DOC)

Information S12 **The identity distributions of SLiMs from IAV PB1 proteins that have differential occurrences in IAVs from different hosts.**
(DOC)

Information S13 **Detail information of comparisons of PB1 protein SLiM compositions from IAVs of different virulence.**
(XLS)

Information S14 **SLiMs that are not highly conserved but appear in HP IAV PB1 proteins.**
(DOC)

Information S15 **Detail information of comparisons of PB2 protein SLiM compositions among IAVs from different hosts.**
(XLS)

Information S16 **Highly conserved SLiMs in IAV PB2 proteins.**
(DOC)

Information S17 **The identity distributions of SLiMs from IAV PB2 proteins that have differential occurrences in IAVs from different hosts.**
(DOC)

Information S18 **Detail information of comparisons of PB2 protein SLiM compositions from IAVs of different virulence.**
(XLS)

Information S19 **SLiMs that are not highly conserved but appear in HP IAV PB2 proteins.**
(DOC)

Information S20 **Detail information of comparisons of NP protein SLiM compositions among IAVs from different hosts.**
(XLS)

Information S21 **Highly conserved SLiMs in IAV NP proteins.**
(DOC)

Information S22 **The identity distributions of SLiMs from IAV PB2 proteins that have differential occurrences in IAVs from different hosts.**
(DOC)

Information S23 **Detail information of comparisons of NP protein SLiM compositions from IAVs of different virulence.**
(XLS)

Information S24 **SLiMs that are not highly conserved but appear in HP IAV NP proteins.**
(DOC)
