# Supplementary material for: A Comparative Study of Short Linear Motif Compositions of the Influenza A Virus Ribonucleoproteins
Source: PLoS One. 2012 Jun 8;7(6):e38637. doi: 10.1371/journal.pone.0038637 (PMC3371030; doi:10.1371/journal.pone.0038637)
Supplement: Information S1 — Number of IAV ribonucleoprotein sequences used in this study. (DOC) [file pone.0038637.s001.doc]

### Table S1. Number of influenza A virus ribonucleoprotein sequences used in this study.

|  | Avian IAVs | | Human IAVs | | Mammal IAVs | |
| --- | --- | --- | --- | --- | --- | --- |
|  | original | used | original | used | original | used |
| PA | 6736 | 4857 | 7584 | 7222 | 1342 | 1061 |
| PB1 | 6755 | 4672 | 7669 | 7164 | 1357 | 1056 |
| PB2 | 6742 | 4271 | 7771 | 7163 | 1293 | 982 |
| NP | 6480 | 5152 | 8105 | 7681 | 1403 | 1224 |
