# Supplementary material for: A Comparative Study of Short Linear Motif Compositions of the Influenza A Virus Ribonucleoproteins
Source: PLoS One. 2012 Jun 8;7(6):e38637. doi: 10.1371/journal.pone.0038637 (PMC3371030; doi:10.1371/journal.pone.0038637)
Supplement: Information S3 — Number of ribonucleoprotein sequences from highly virulent/pandemic IAVs used in this study. (DOC) [file pone.0038637.s003.doc]

Table S3. Number of RNP sequences from highly virulent/pandemic IAVs used in this study.

|  | Pan_PA | Pan_PB1 | Pan_PB2 | Pan_NP |
| --- | --- | --- | --- | --- |
| H1N1 1918 | 1 | 1 | 1 | 1 |
| H1N1 1977 Russia | 3 | 3 | 3 | 4 |
| H1N1 2009 | 30 | 33 | 29 | 39 |
| H2N2 1957 | 20 | 20 | 20 | 19 |
| H3N2 1968 | 33 | 33 | 33 | 35 |
| H5N1 1997 Hong Kong | 18 | 16 | 15 | 19 |
| H5N1 Indonesia | 71 | 69 | 69 | 86 |
| H5N1 Thailand | 12 | 13 | 14 | 14 |
| H5N1 Viet Nam | 31 | 34 | 34 | 46 |
| Total | 219 | 222 | 141 | 263 |
