# Supplementary material for: A Comparative Study of Short Linear Motif Compositions of the Influenza A Virus Ribonucleoproteins
Source: PLoS One. 2012 Jun 8;7(6):e38637. doi: 10.1371/journal.pone.0038637 (PMC3371030; doi:10.1371/journal.pone.0038637)
Supplement: Information S7 — The identity distributions of SLiMs from IAV PA proteins that have differential occurrences in IAVs from different hosts. (DOC) [file pone.0038637.s007.doc]

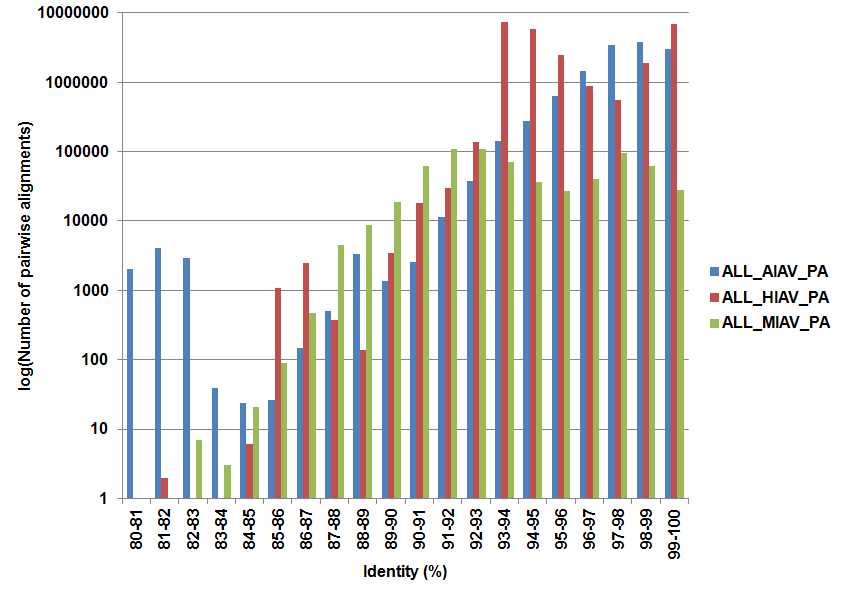


PA Identity Distribution 1. The distribution of pairwise alignment identity of all PA protein sequences from avian, human and mammalian IAVs. The x-axis is the number of pairwise alignments of IAV PA protein sequences. The y-axis is the identity of pairwise alignment (the percentage of identical amino acids that are the same in both PA sequences). Blue: PA protein sequences from avian IAVs. Red: PA protein sequences from human IAVs. Green: PA protein sequences from mammalian IAVs.


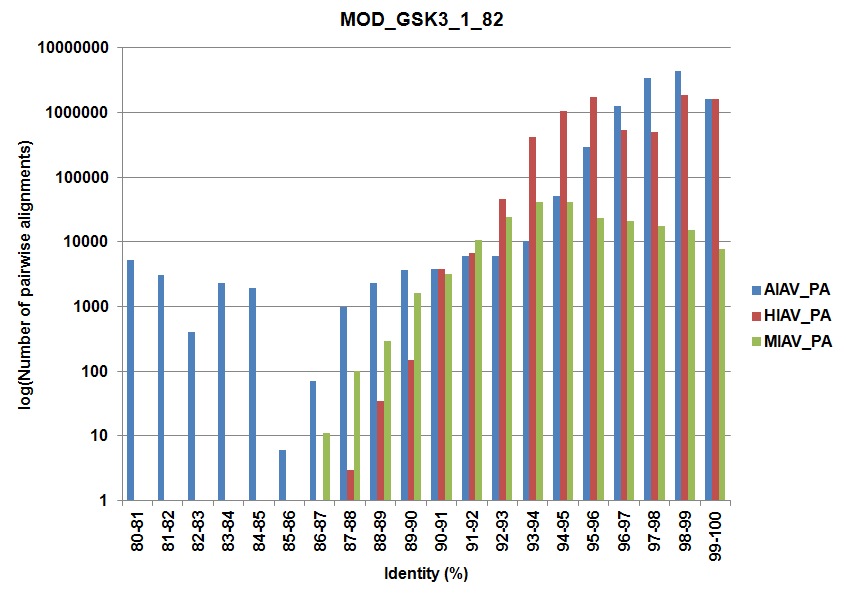


PA Identity Distribution 2. The distribution of pairwise alignment identity of PA protein sequences which harbor the SLiM MOD_GSK3_1_82 from avian, human and mammalian IAVs. The x-axis is the number of pairwise alignments of IAV PA protein sequences. The y-axis is the identity of pairwise alignment (the percentage of identical amino acids that are the same in both PA sequences). Blue: PA protein sequences from avian IAVs. Red: PA protein sequences from human IAVs. Green: PA protein sequences from mammalian IAVs.


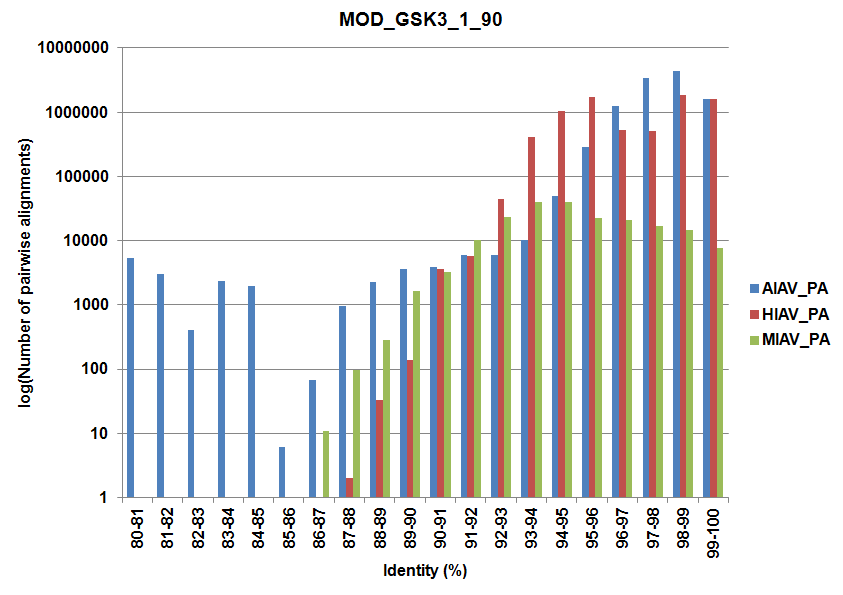


PA Identity Distribution 3. The distribution of pairwise alignment identity of PA protein sequences which harbor the SLiM MOD_GSK3_1_90 from avian, human and mammalian IAVs. The x-axis is the number of pairwise alignments of IAV PA protein sequences. The y-axis is the identity of pairwise alignment (the percentage of identical amino acids that are the same in both PA sequences). Blue: PA protein sequences from avian IAVs. Red: PA protein sequences from human IAVs. Green: PA protein sequences from mammalian IAVs.


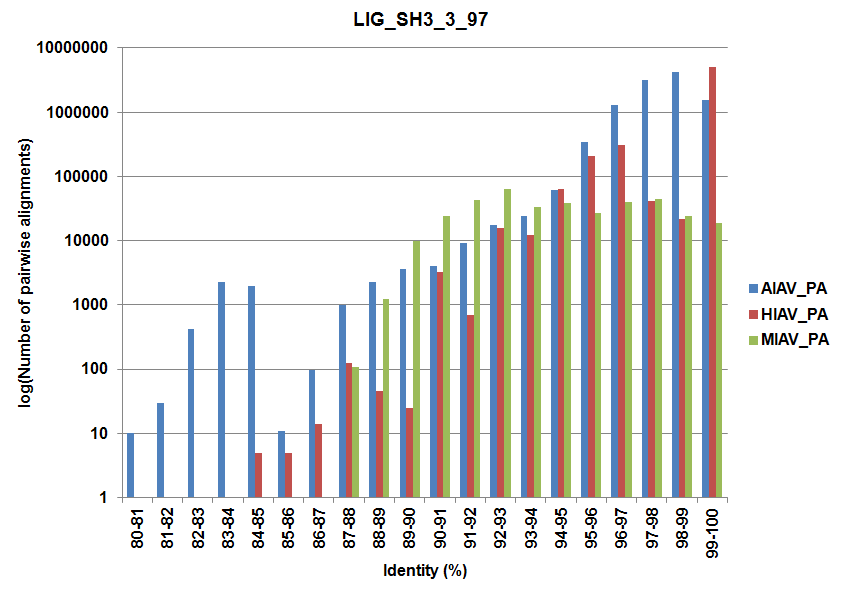


PA Identity Distribution 4. The distribution of pairwise alignment identity of PA protein sequences which harbor the SLiM LIG_SH3_1_97 from avian, human and mammalian IAVs. The x-axis is the number of pairwise alignments of IAV PA protein sequences. The y-axis is the identity of pairwise alignment (the percentage of identical amino acids that are the same in both PA sequences). Blue: PA protein sequences from avian IAVs. Red: PA protein sequences from human IAVs. Green: PA protein sequences from mammalian IAVs.


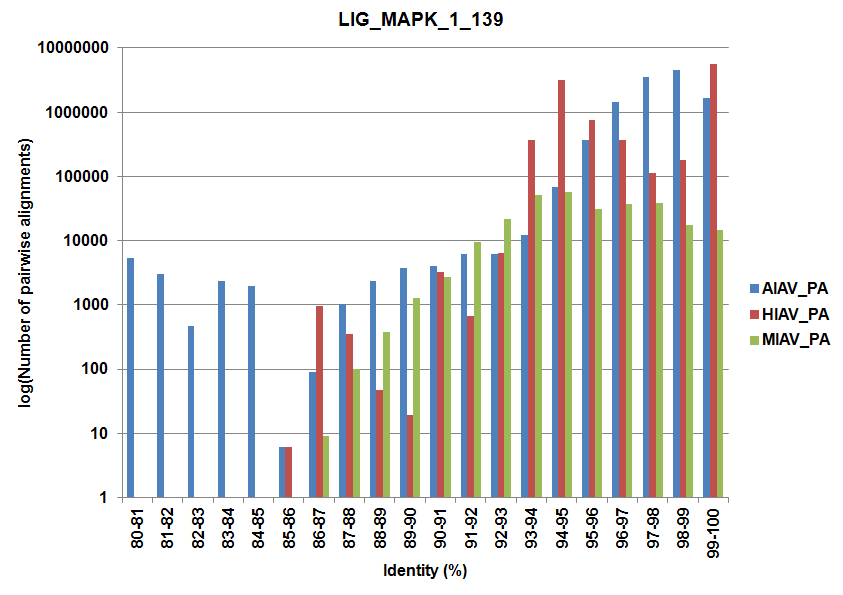


PA Identity Distribution 5. The distribution of pairwise alignment identity of PA protein sequences which harbor the SLiM LIG_MAPK_1_139 from avian, human and mammalian IAVs. The x-axis is the number of pairwise alignments of IAV PA protein sequences. The y-axis is the identity of pairwise alignment (the percentage of identical amino acids that are the same in both PA sequences). Blue: PA protein sequences from avian IAVs. Red: PA protein sequences from human IAVs. Green: PA protein sequences from mammalian IAVs.


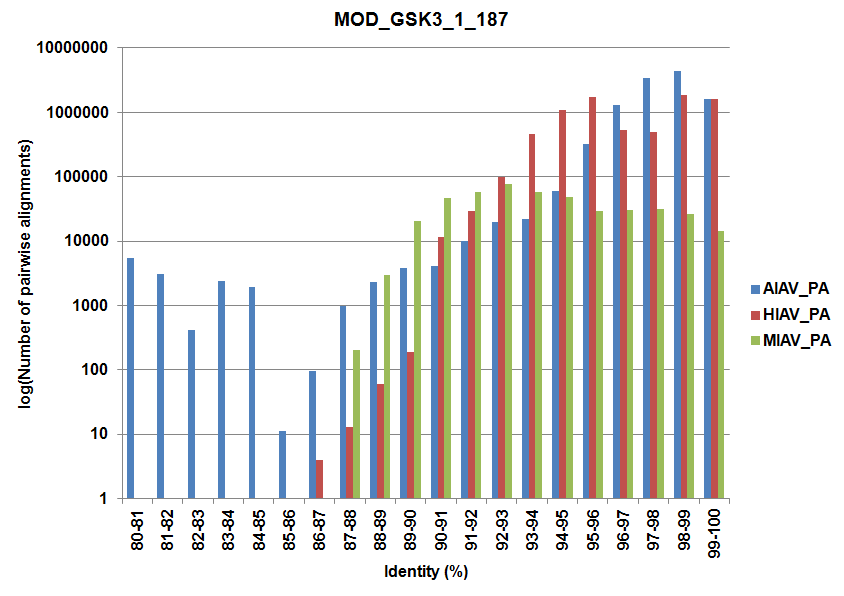


PA Identity Distribution 6. The distribution of pairwise alignment identity of PA protein sequences which harbor the SLiM MOD_GSK3_1_187 from avian, human and mammalian IAVs. The x-axis is the number of pairwise alignments of IAV PA protein sequences. The y-axis is the identity of pairwise alignment (the percentage of identical amino acids that are the same in both PA sequences). Blue: PA protein sequences from avian IAVs. Red: PA protein sequences from human IAVs. Green: PA protein sequences from mammalian IAVs.


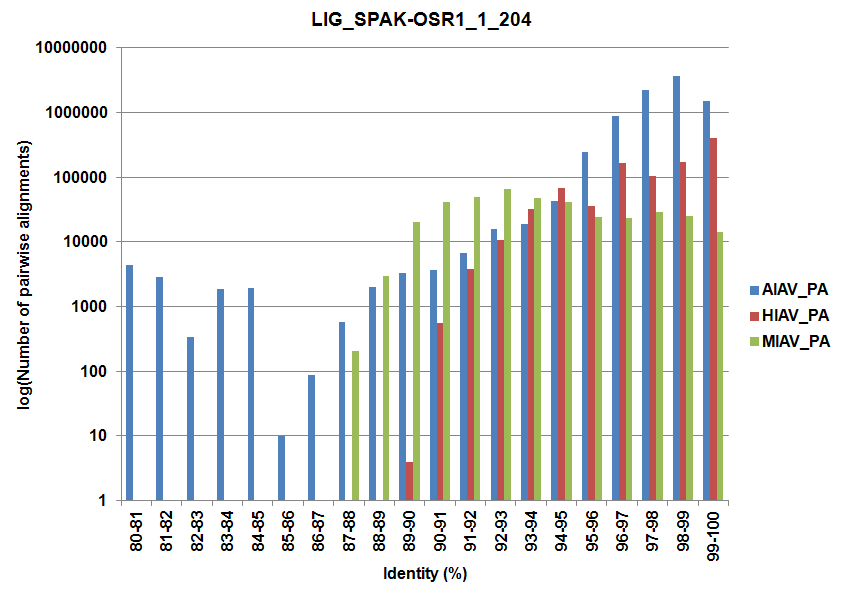


PA Identity Distribution 7. The distribution of pairwise alignment identity of PA protein sequences which harbor the SLiM LIG_SPAK-OSR1_1_204 from avian, human and mammalian IAVs. The x-axis is the number of pairwise alignments of IAV PA protein sequences. The y-axis is the identity of pairwise alignment (the percentage of identical amino acids that are the same in both PA sequences). Blue: PA protein sequences from avian IAVs. Red: PA protein sequences from human IAVs. Green: PA protein sequences from mammalian IAVs.


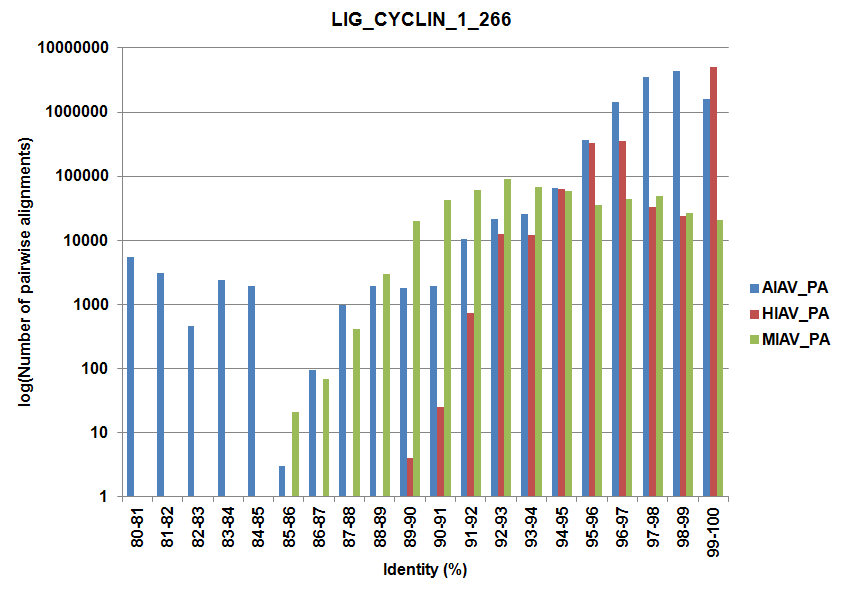


PA Identity Distribution 8. The distribution of pairwise alignment identity of PA protein sequences which harbor the SLiM LIG_CYCLIN_1_266 from avian, human and mammalian IAVs. The x-axis is the number of pairwise alignments of IAV PA protein sequences. The y-axis is the identity of pairwise alignment (the percentage of identical amino acids that are the same in both PA sequences). Blue: PA protein sequences from avian IAVs. Red: PA protein sequences from human IAVs. Green: PA protein sequences from mammalian IAVs.


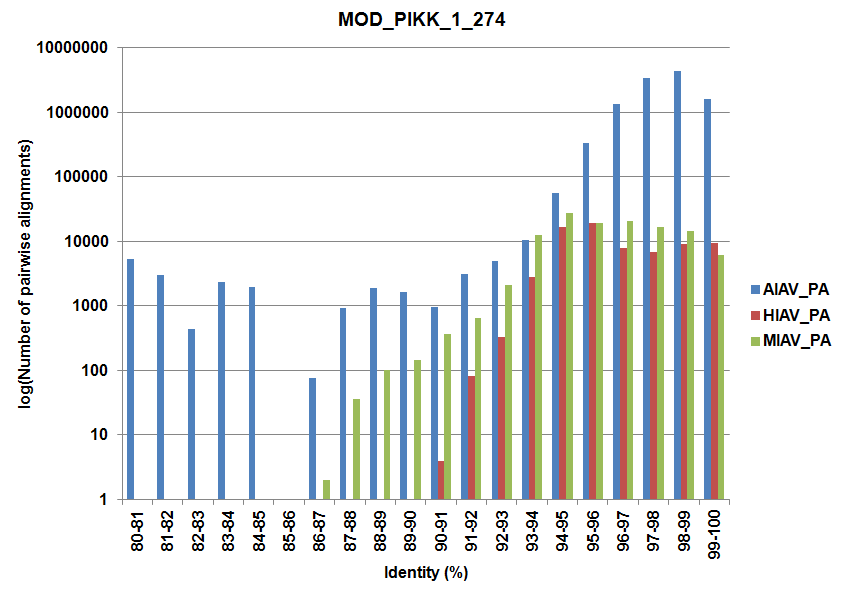


PA Identity Distribution 9. The distribution of pairwise alignment identity of PA protein sequences which harbor the SLiM MOD_PIKK_1_274 from avian, human and mammalian IAVs. The x-axis is the number of pairwise alignments of IAV PA protein sequences. The y-axis is the identity of pairwise alignment (the percentage of identical amino acids that are the same in both PA sequences). Blue: PA protein sequences from avian IAVs. Red: PA protein sequences from human IAVs. Green: PA protein sequences from mammalian IAVs.


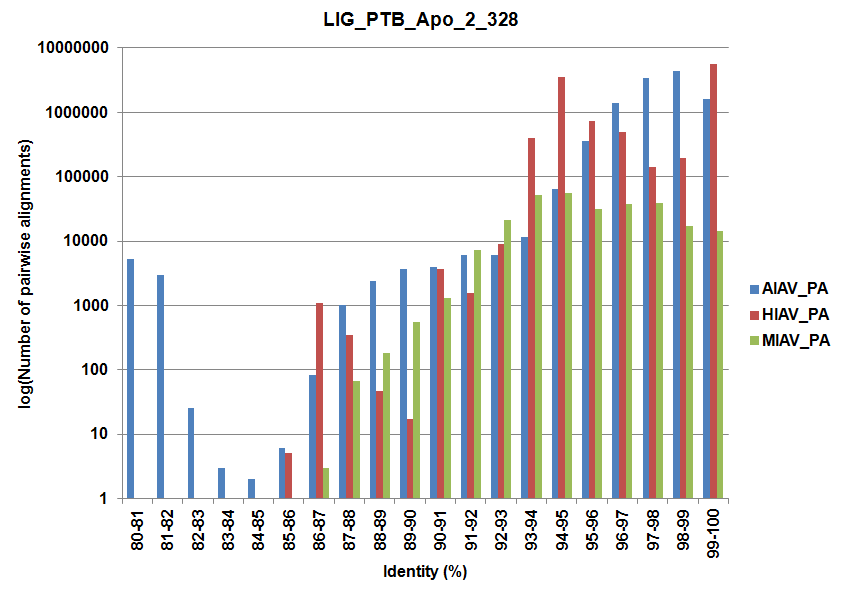


PA Identity Distribution 10. The distribution of pairwise alignment identity of PA protein sequences which harbor the SLiM LIG_PTB_Apo_2_328 from avian, human and mammalian IAVs. The x-axis is the number of pairwise alignments of IAV PA protein sequences. The y-axis is the identity of pairwise alignment (the percentage of identical amino acids that are the same in both PA sequences). Blue: PA protein sequences from avian IAVs. Red: PA protein sequences from human IAVs. Green: PA protein sequences from mammalian IAVs.


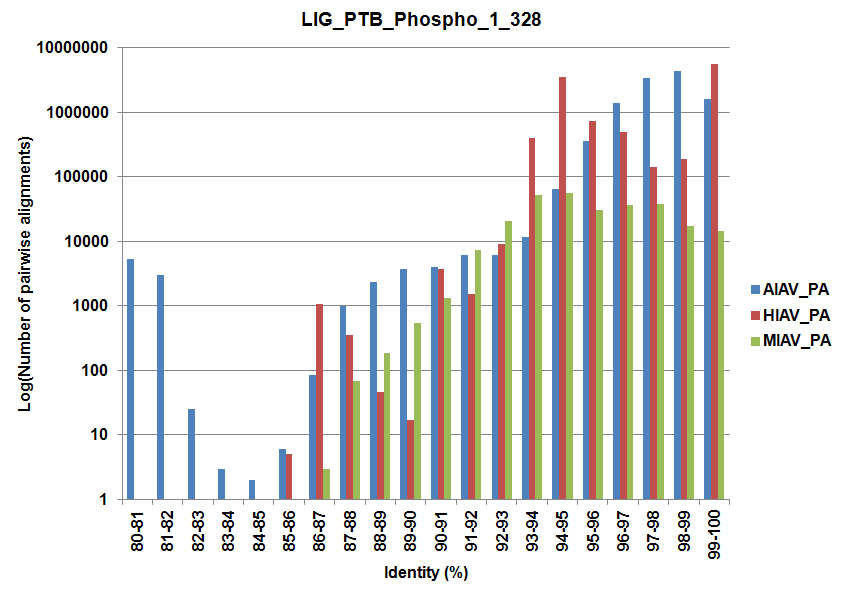


PA Identity Distribution 11. The distribution of pairwise alignment identity of PA protein sequences which harbor the SLiM LIG_PTB_Phospho_1_328 from avian, human and mammalian IAVs. The x-axis is the number of pairwise alignments of IAV PA protein sequences. The y-axis is the identity of pairwise alignment (the percentage of identical amino acids that are the same in both PA sequences). Blue: PA protein sequences from avian IAVs. Red: PA protein sequences from human IAVs. Green: PA protein sequences from mammalian IAVs.


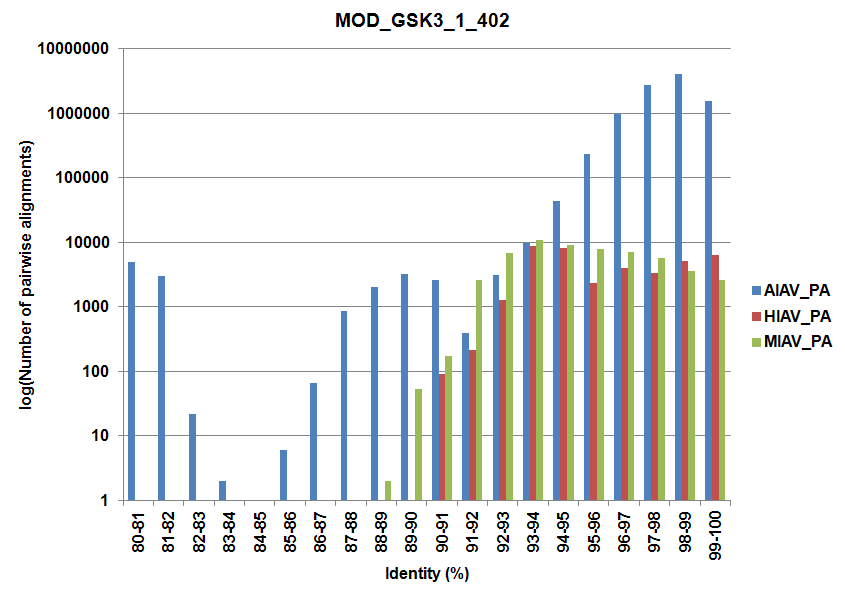


PA Identity Distribution 12. The distribution of pairwise alignment identity of PA protein sequences which harbor the SLiM MOD_GSK3_1_402 from avian, human and mammalian IAVs. The x-axis is the number of pairwise alignments of IAV PA protein sequences. The y-axis is the identity of pairwise alignment (the percentage of identical amino acids that are the same in both PA sequences). Blue: PA protein sequences from avian IAVs. Red: PA protein sequences from human IAVs. Green: PA protein sequences from mammalian IAVs.


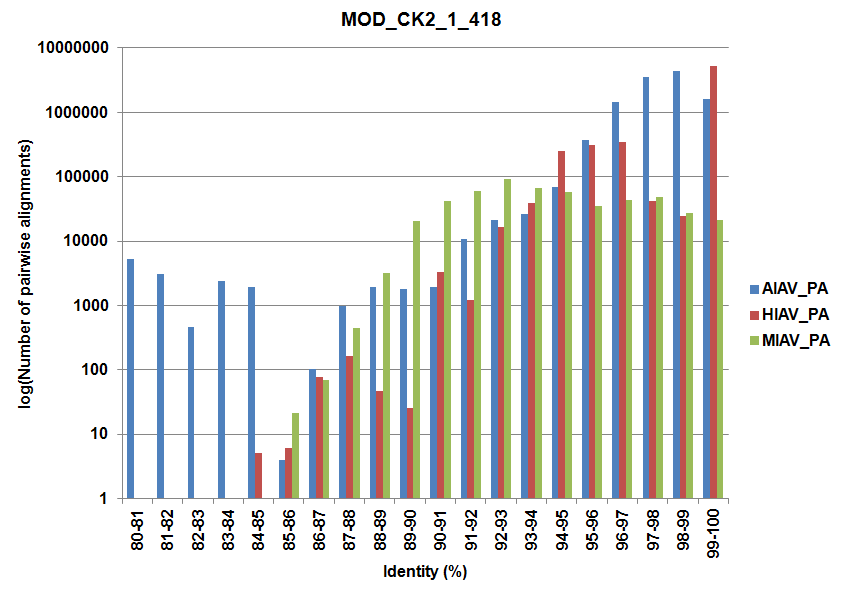


PA Identity Distribution 13. The distribution of pairwise alignment identity of PA protein sequences which harbor the SLiM MOD_CK3_1_418 from avian, human and mammalian IAVs. The x-axis is the number of pairwise alignments of IAV PA protein sequences. The y-axis is the identity of pairwise alignment (the percentage of identical amino acids that are the same in both PA sequences). Blue: PA protein sequences from avian IAVs. Red: PA protein sequences from human IAVs. Green: PA protein sequences from mammalian IAVs.


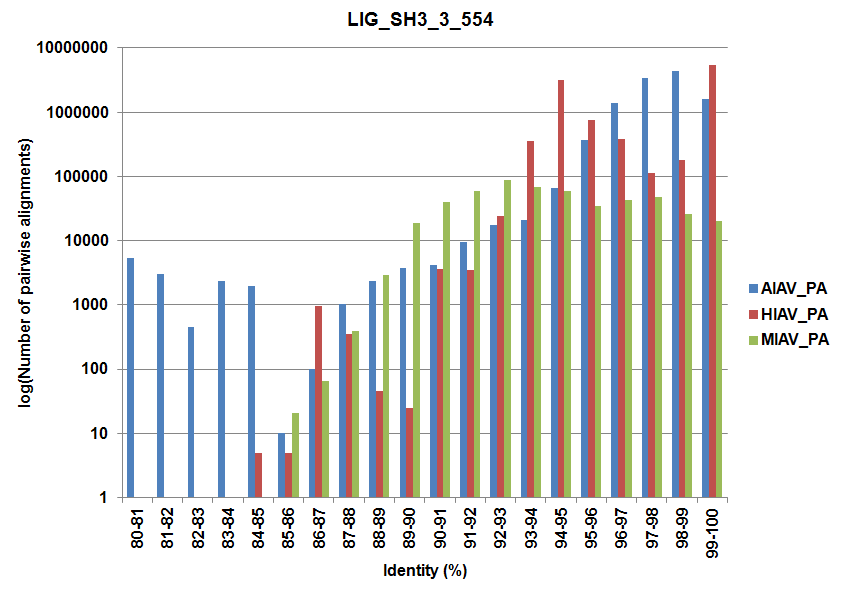


PA Identity Distribution 14. The distribution of pairwise alignment identity of PA protein sequences which harbor the SLiM LIG_SH3_3_554 from avian, human and mammalian IAVs. The x-axis is the number of pairwise alignments of IAV PA protein sequences. The y-axis is the identity of pairwise alignment (the percentage of identical amino acids that are the same in both PA sequences). Blue: PA protein sequences from avian IAVs. Red: PA protein sequences from human IAVs. Green: PA protein sequences from mammalian IAVs.


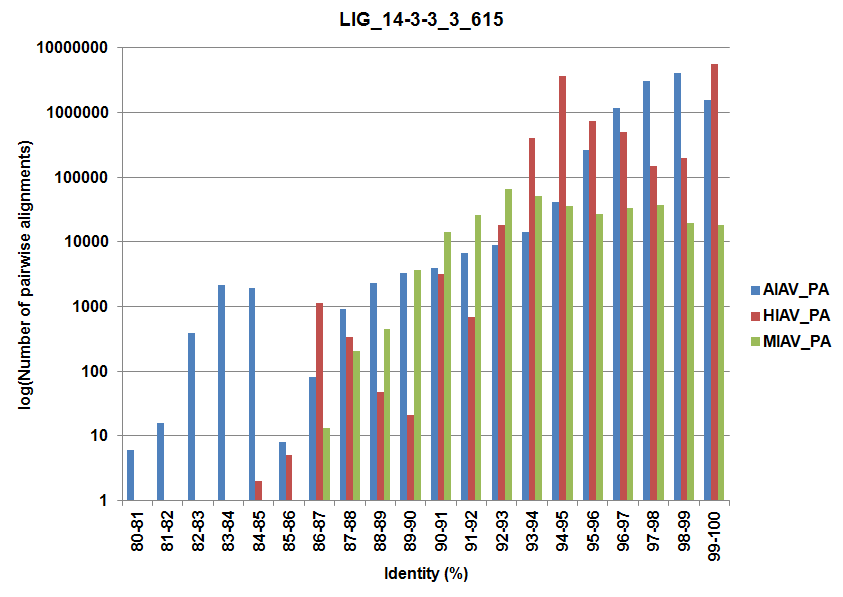


PA Identity Distribution 15. The distribution of pairwise alignment identity of PA protein sequences which harbor the SLiM LIG_14-3-3_3_615 from avian, human and mammalian IAVs. The x-axis is the number of pairwise alignments of IAV PA protein sequences. The y-axis is the identity of pairwise alignment (the percentage of identical amino acids that are the same in both PA sequences). Blue: PA protein sequences from avian IAVs. Red: PA protein sequences from human IAVs. Green: PA protein sequences from mammalian IAVs.
