# Supplementary material for: A Comparative Study of Short Linear Motif Compositions of the Influenza A Virus Ribonucleoproteins
Source: PLoS One. 2012 Jun 8;7(6):e38637. doi: 10.1371/journal.pone.0038637 (PMC3371030; doi:10.1371/journal.pone.0038637)
Supplement: Information S9 — SLiMs that are not highly conserved but appear in virulent/pandemic IAV PA proteins. (DOC) [file pone.0038637.s009.doc]

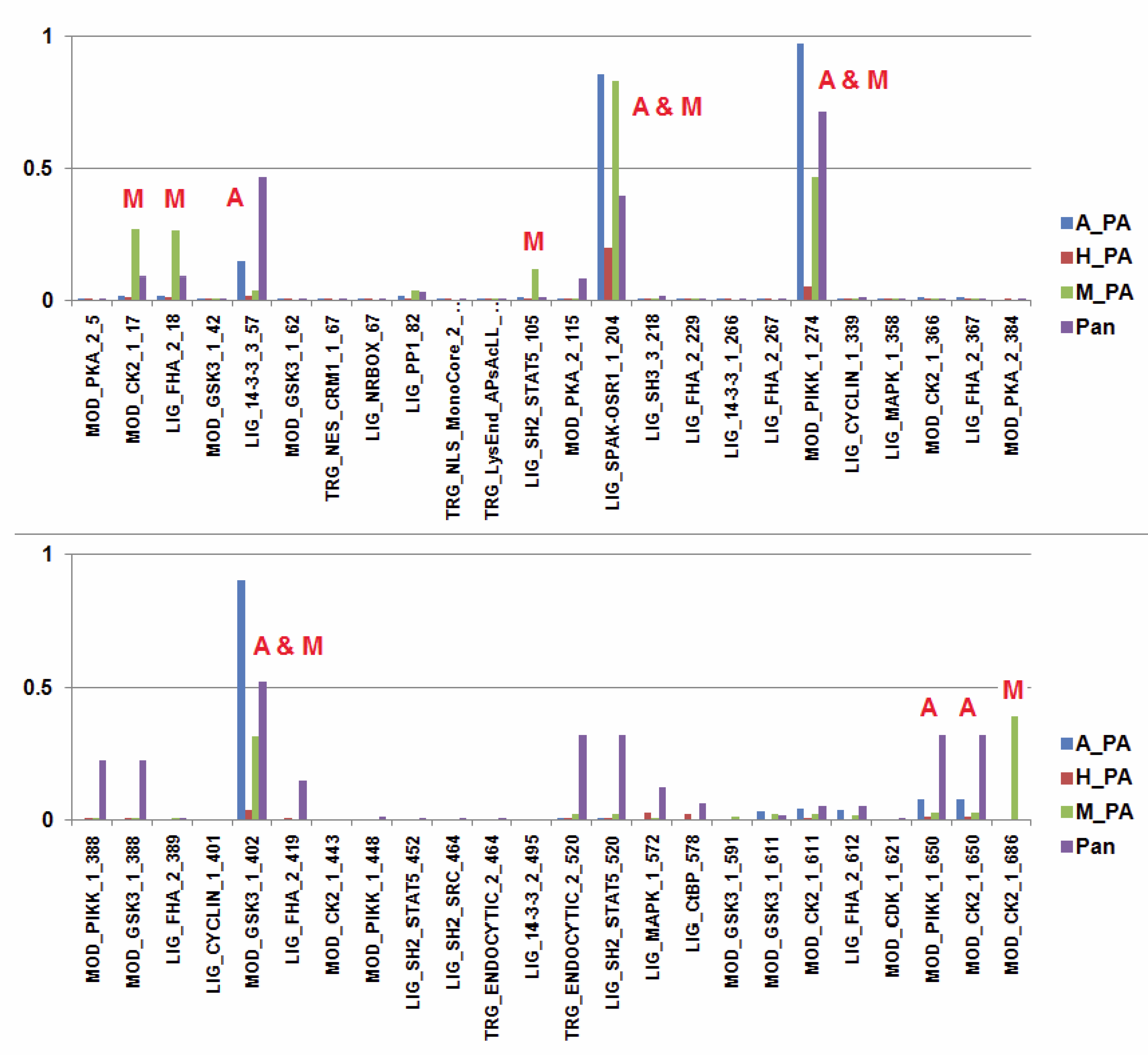


## Figure S9. SLiMs that are not highly conserved but appear in HP IAV PA proteins.

The Y-axis indicates the occurrence of each identified SLiM. The X-axis indicates the name and position of each identified SLiM in the PA proteins. For example, “MOD_PKA_2” in “MOD_PKA_2_5” is the name of the SLiM, and 5 is the amino acid position where the SLiM starts. A_PA, H_PA and M_PA indicate the PA proteins from avian, human and IAV, respectively. Pan indicates PA proteins from highly virulent/pandemic IAVs. The red label “A” indicates an avian IAV specific SLiM. The red label “M” indicates a mammalian IAV specific SLiM. The red label “A&M” indicates an avian and mammalian IAV specific SLiM.
