# Supplementary material for: A Comparative Study of Short Linear Motif Compositions of the Influenza A Virus Ribonucleoproteins
Source: PLoS One. 2012 Jun 8;7(6):e38637. doi: 10.1371/journal.pone.0038637 (PMC3371030; doi:10.1371/journal.pone.0038637)
Supplement: Information S12 — The identity distributions of SLiMs from IAV PB1 proteins that have differential occurrences in IAVs from different hosts. (DOC) [file pone.0038637.s012.doc]

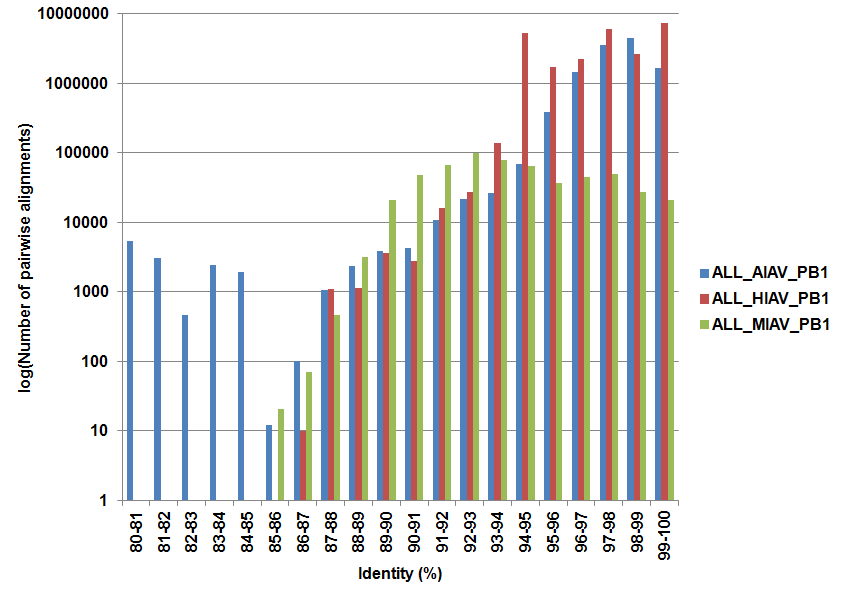


PB1 Identity Distribution 1. The distribution of pairwise alignment identity of all PB1 protein sequences from avian, human and mammalian IAVs. The x-axis is the number of pairwise alignments of IAV PB1 protein sequences. The y-axis is the identity of pairwise alignment (the percentage of identical amino acids that are the same in both PB1 sequences). Blue: PB1 protein sequences from avian IAVs. Red: PB1 protein sequences from human IAVs. Green: PB1 protein sequences from mammalian IAVs.


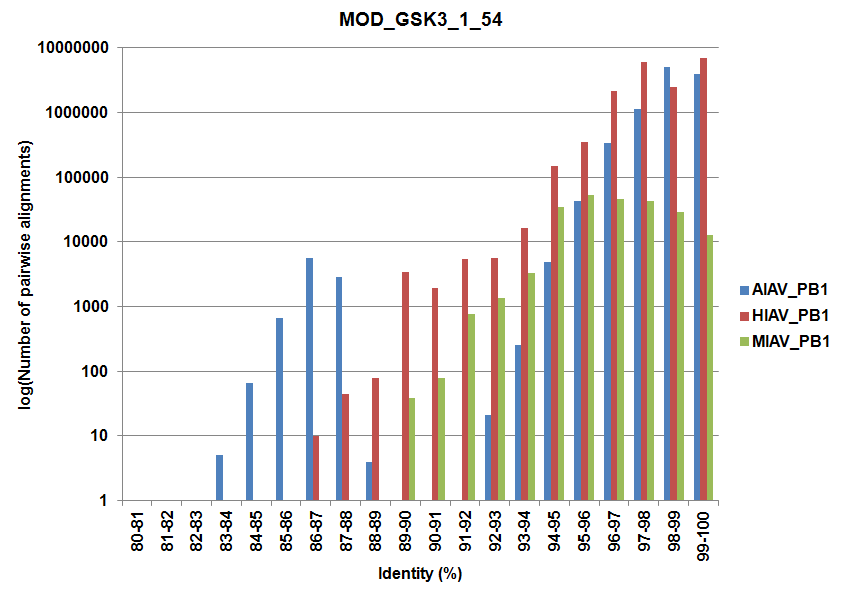


PB1 Identity Distribution 2. The distribution of pairwise alignment identity of PB1 protein sequences which harbor the SLiM MOD_GSK3_1_54 from avian, human and mammalian IAVs. The x-axis is the number of pairwise alignments of IAV PB1 protein sequences. The y-axis is the identity of pairwise alignment (the percentage of identical amino acids that are the same in both PB1 sequences). Blue: PB1 protein sequences from avian IAVs. Red: PB1 protein sequences from human IAVs. Green: PB1 protein sequences from mammalian IAVs.


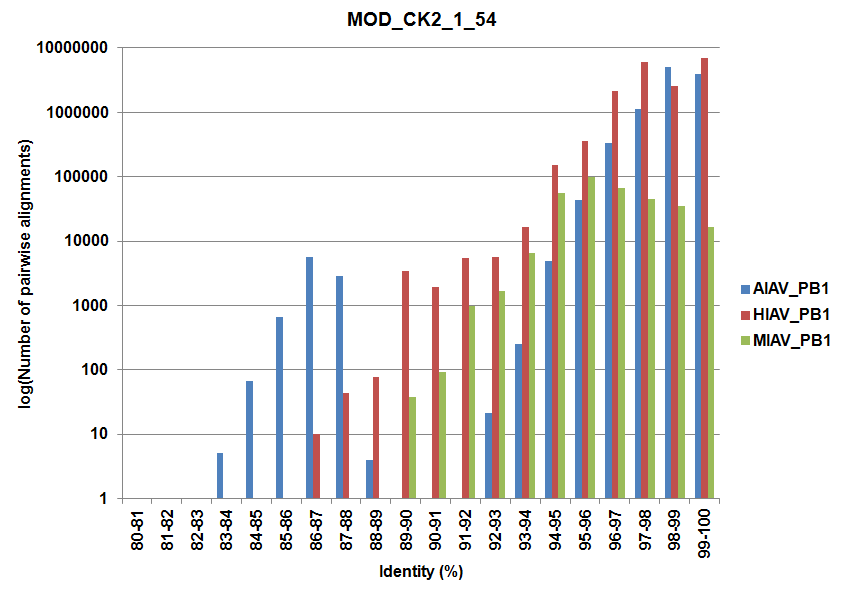


PB1 Identity Distribution 3. The distribution of pairwise alignment identity of PB1 protein sequences which harbor the SLiM MOD_CK2_1_54 from avian, human and mammalian IAVs. The x-axis is the number of pairwise alignments of IAV PB1 protein sequences. The y-axis is the identity of pairwise alignment (the percentage of identical amino acids that are the same in both PB1 sequences). Blue: PB1 protein sequences from avian IAVs. Red: PB1 protein sequences from human IAVs. Green: PB1 protein sequences from mammalian IAVs.


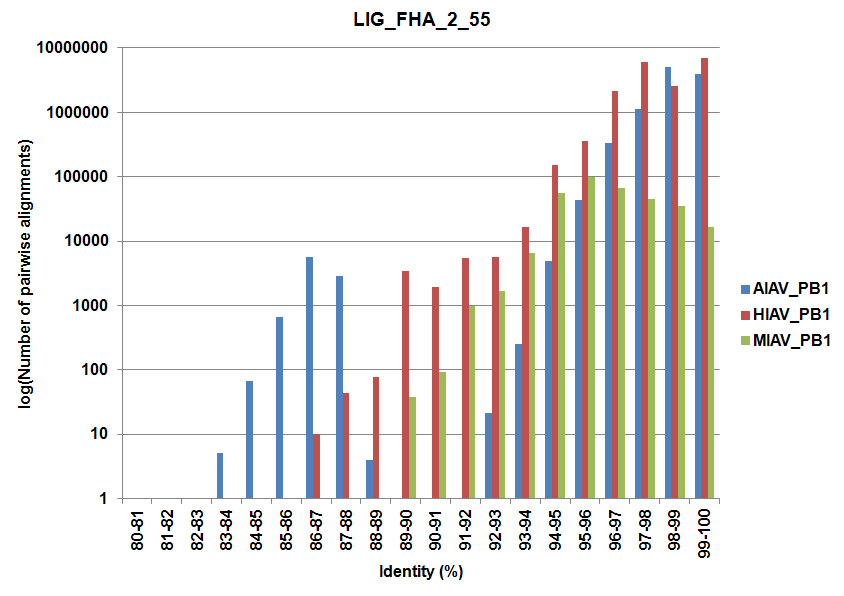


PB1 Identity Distribution 4. The distribution of pairwise alignment identity of PB1 protein sequences which harbor the SLiM LIG_FHA_2_55 from avian, human and mammalian IAVs. The x-axis is the number of pairwise alignments of IAV PB1 protein sequences. The y-axis is the identity of pairwise alignment (the percentage of identical amino acids that are the same in both PB1 sequences). Blue: PB1 protein sequences from avian IAVs. Red: PB1 protein sequences from human IAVs. Green: PB1 protein sequences from mammalian IAVs.


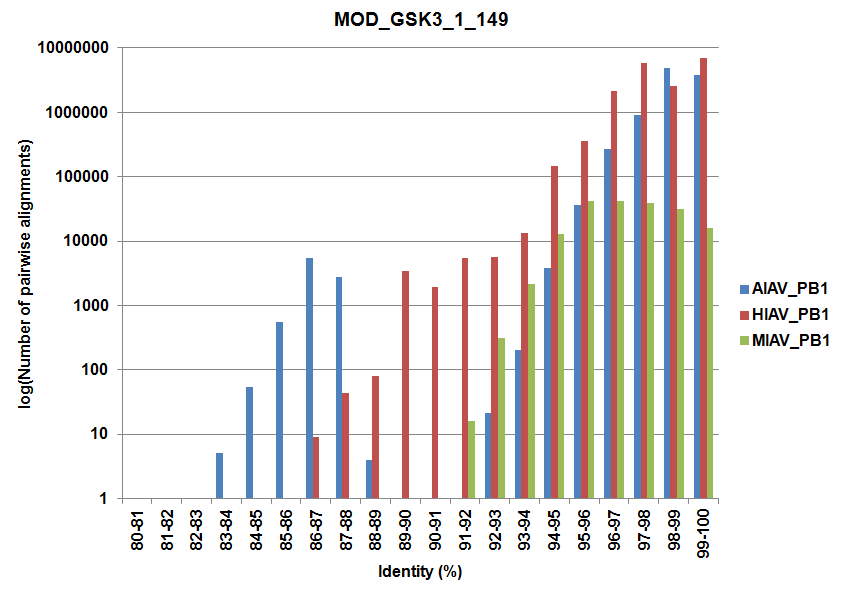


PB1 Identity Distribution 5. The distribution of pairwise alignment identity of PB1 protein sequences which harbor the SLiM MOD_GSK3_1_149 from avian, human and mammalian IAVs. The x-axis is the number of pairwise alignments of IAV PB1 protein sequences. The y-axis is the identity of pairwise alignment (the percentage of identical amino acids that are the same in both PB1 sequences). Blue: PB1 protein sequences from avian IAVs. Red: PB1 protein sequences from human IAVs. Green: PB1 protein sequences from mammalian IAVs.


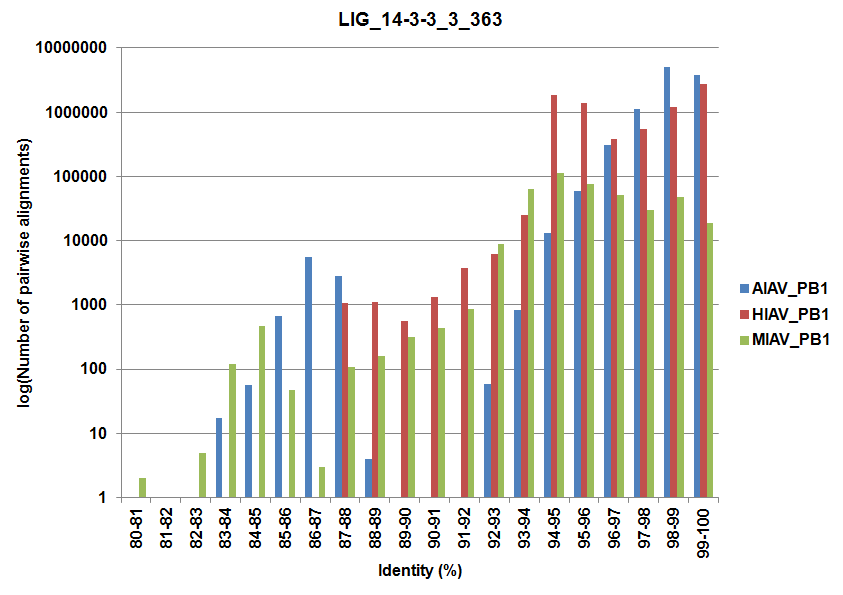


PB1 Identity Distribution 6. The distribution of pairwise alignment identity of PB1 protein sequences which harbor the SLiM LIG_14-3-3_3_363 from avian, human and mammalian IAVs. The x-axis is the number of pairwise alignments of IAV PB1 protein sequences. The y-axis is the identity of pairwise alignment (the percentage of identical amino acids that are the same in both PB1 sequences). Blue: PB1 protein sequences from avian IAVs. Red: PB1 protein sequences from human IAVs. Green: PB1 protein sequences from mammalian IAVs.


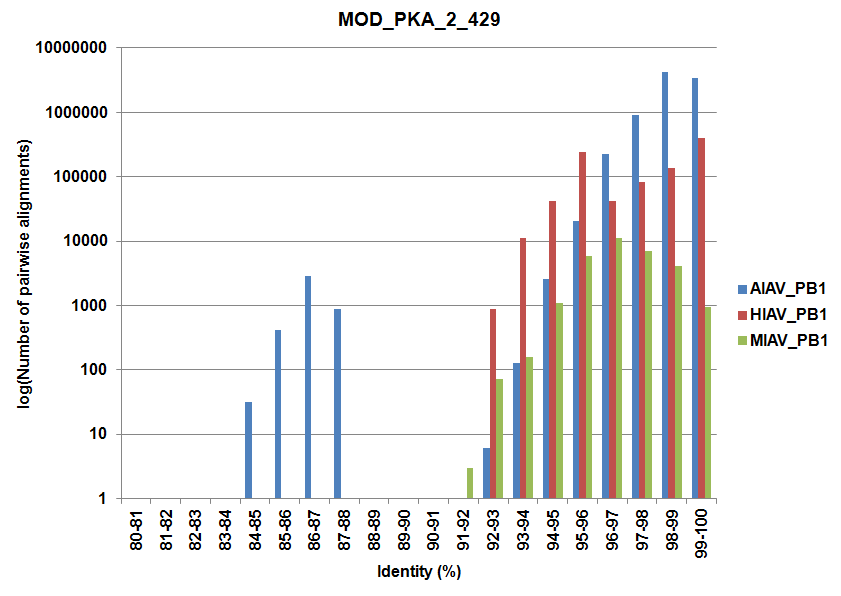


PB1 Identity Distribution 7. The distribution of pairwise alignment identity of PB1 protein sequences which harbor the SLiM MOD_PKA_2_429 from avian, human and mammalian IAVs. The x-axis is the number of pairwise alignments of IAV PB1 protein sequences. The y-axis is the identity of pairwise alignment (the percentage of identical amino acids that are the same in both PB1 sequences). Blue: PB1 protein sequences from avian IAVs. Red: PB1 protein sequences from human IAVs. Green: PB1 protein sequences from mammalian IAVs.


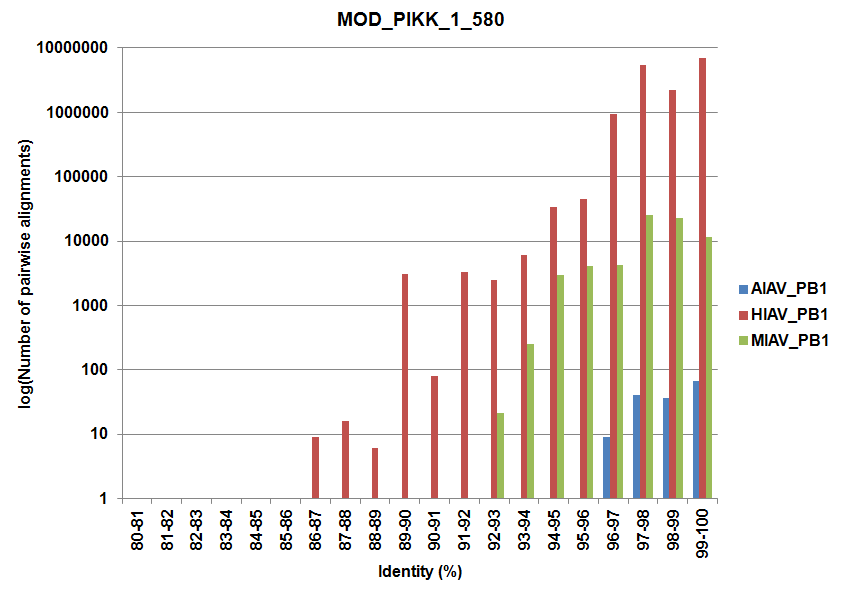


PB1 Identity Distribution 8. The distribution of pairwise alignment identity of PB1 protein sequences which harbor the SLiM MOD_PIKK_1_580 from avian, human and mammalian IAVs. The x-axis is the number of pairwise alignments of IAV PB1 protein sequences. The y-axis is the identity of pairwise alignment (the percentage of identical amino acids that are the same in both PB1 sequences). Blue: PB1 protein sequences from avian IAVs. Red: PB1 protein sequences from human IAVs. Green: PB1 protein sequences from mammalian IAVs.


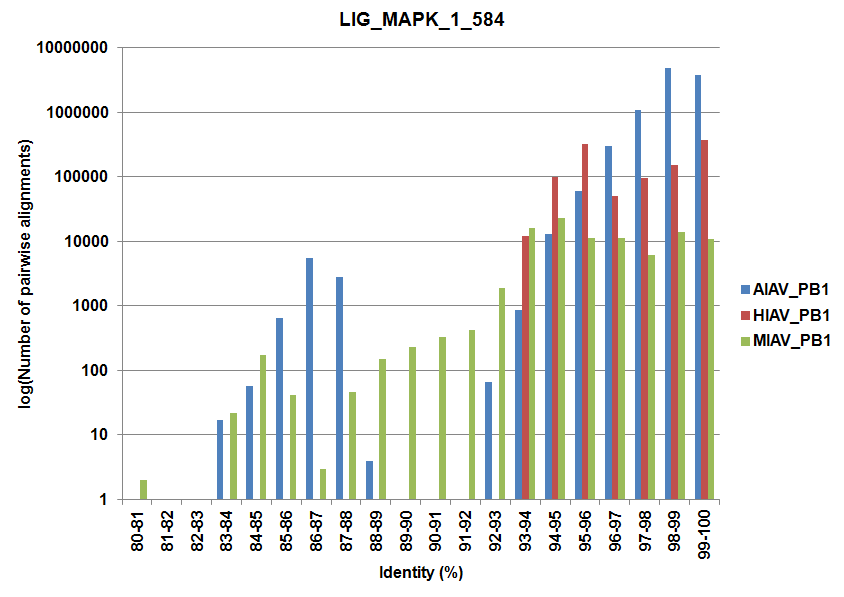


PB1 Identity Distribution 9. The distribution of pairwise alignment identity of PB1 protein sequences which harbor the SLiM LIG_MAPK_1_584 from avian, human and mammalian IAVs. The x-axis is the number of pairwise alignments of IAV PB1 protein sequences. The y-axis is the identity of pairwise alignment (the percentage of identical amino acids that are the same in both PB1 sequences). Blue: PB1 protein sequences from avian IAVs. Red: PB1 protein sequences from human IAVs. Green: PB1 protein sequences from mammalian IAVs.


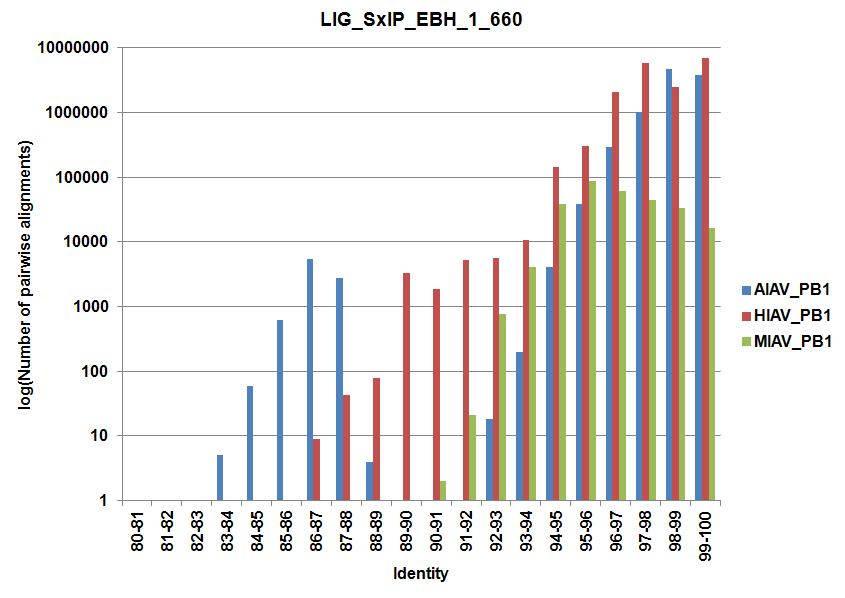


PB1 Identity Distribution 10. The distribution of pairwise alignment identity of PB1 protein sequences which harbor the SLiM LIG_SxIP_EBH_1_660 from avian, human and mammalian IAVs. The x-axis is the number of pairwise alignments of IAV PB1 protein sequences. The y-axis is the identity of pairwise alignment (the percentage of identical amino acids that are the same in both PB1 sequences). Blue: PB1 protein sequences from avian IAVs. Red: PB1 protein sequences from human IAVs. Green: PB1 protein sequences from mammalian IAVs.
