# Supplementary material for: A Comparative Study of Short Linear Motif Compositions of the Influenza A Virus Ribonucleoproteins
Source: PLoS One. 2012 Jun 8;7(6):e38637. doi: 10.1371/journal.pone.0038637 (PMC3371030; doi:10.1371/journal.pone.0038637)
Supplement: Information S14 — SLiMs that are not highly conserved but appear in HP IAV PB1 proteins. (DOC) [file pone.0038637.s014.doc]

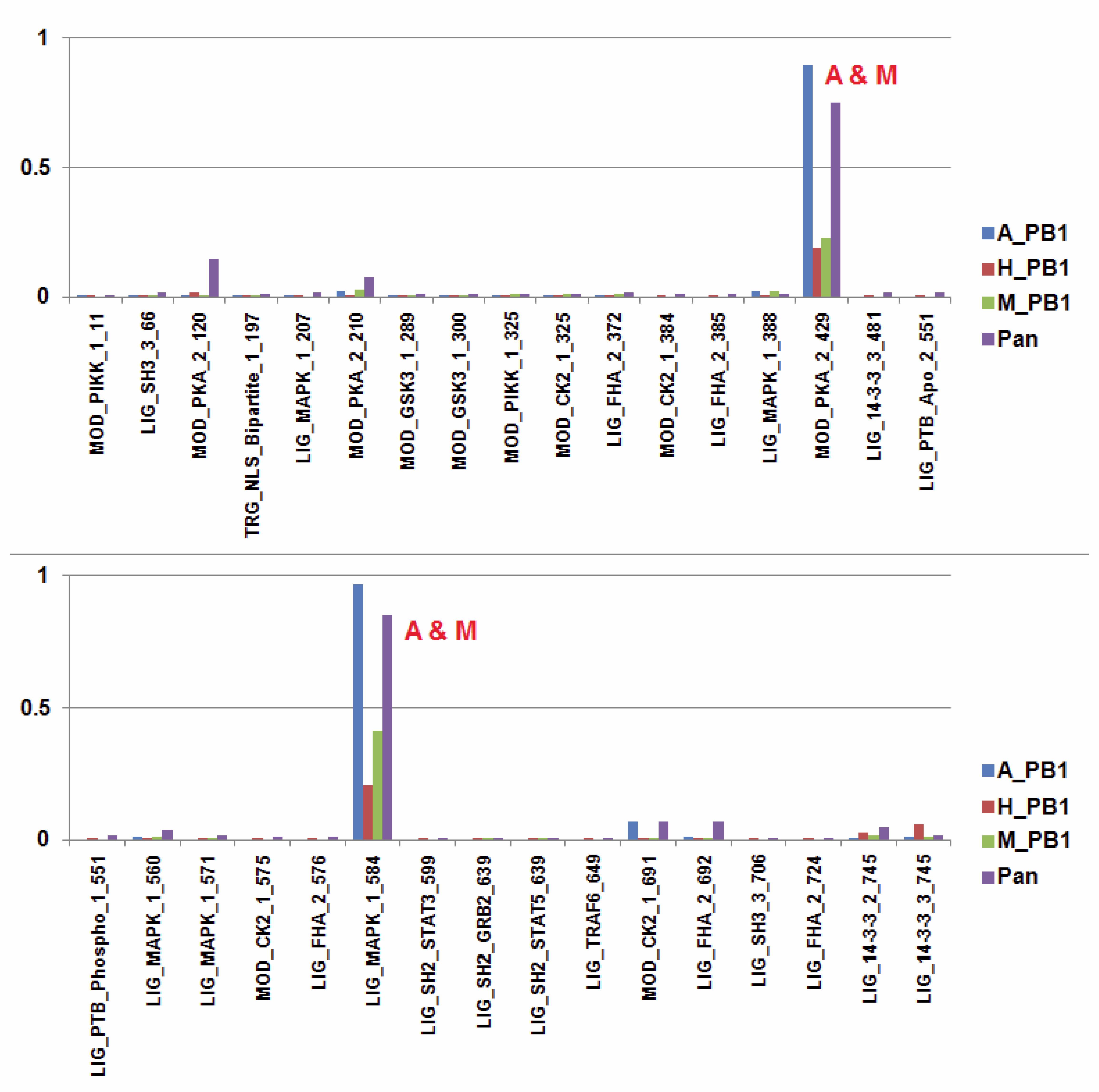


## Figure S14. SLiMs that are not highly conserved but appear in HP IAV PB1 proteins.

The Y-axis indicates the occurrence of each identified SLiM. The X-axis indicates the name and position of each identified SLiM in the PB1 proteins. For example, “MOD_PIKK_1” in “MOD_PIKK_1_11” is the name of the SLiM, and 11 is the amino acid position where the SLiM starts. A_PB1, H_PB1 and M_PB1 indicate the PB1 proteins from avian, human and IAV, respectively. Pan indicates PB1 proteins from highly virulent/pandemic IAVs. The red label “A” indicates an avian IAV specific SLiM. The red label “M” indicates a mammalian IAV specific SLiM. The red label “A&M” indicates an avian and mammalian IAV specific SLiM.
