# Supplementary material for: A Comparative Study of Short Linear Motif Compositions of the Influenza A Virus Ribonucleoproteins
Source: PLoS One. 2012 Jun 8;7(6):e38637. doi: 10.1371/journal.pone.0038637 (PMC3371030; doi:10.1371/journal.pone.0038637)
Supplement: Information S17 — The identity distributions of SLiMs from IAV PB2 proteins that have differential occurrences in IAVs from different hosts. (DOC) [file pone.0038637.s017.doc]

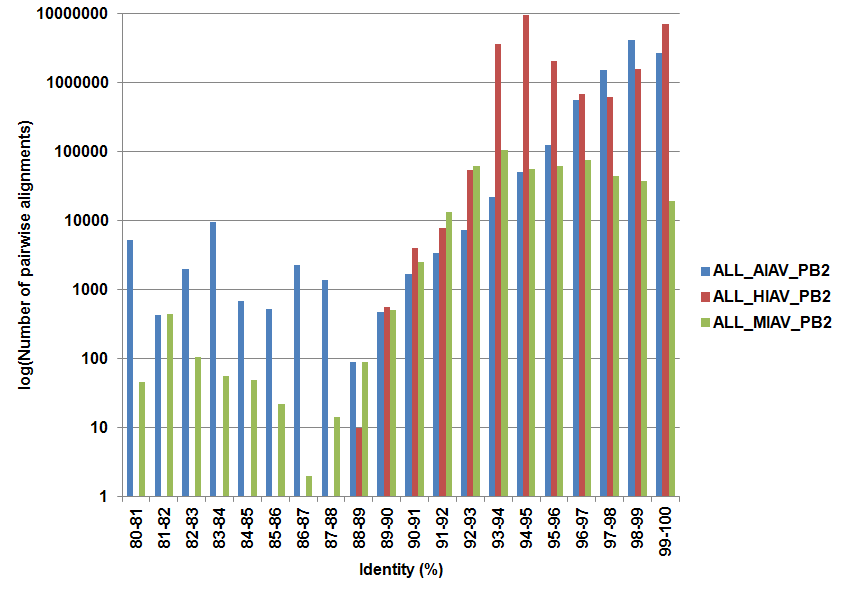


PB2 Identity Distribution 1. The distribution of pairwise alignment identity of all PB2 protein sequences from avian, human and mammalian IAVs. The x-axis is the number of pairwise alignments of IAV PB2 protein sequences. The y-axis is the identity of pairwise alignment (the percentage of identical amino acids that are the same in both PB2 sequences). Blue: PB2 protein sequences from avian IAVs. Red: PB2 protein sequences from human IAVs. Green: PB2 protein sequences from mammalian IAVs.


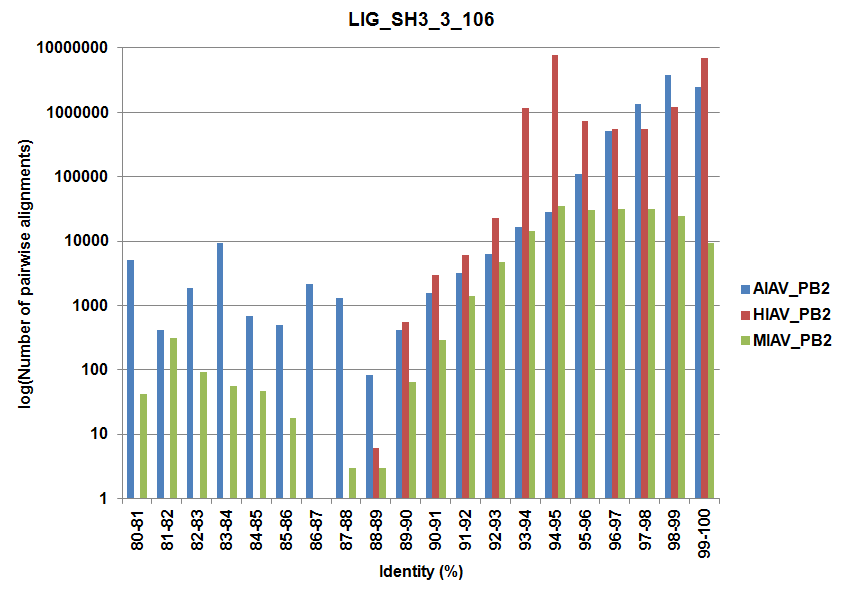


PB2 Identity Distribution 2. The distribution of pairwise alignment identity of PB2 protein sequences which harbor the SLiM LIG_SH3_3_106 from avian, human and mammalian IAVs. The x-axis is the number of pairwise alignments of IAV PB2 protein sequences. The y-axis is the identity of pairwise alignment (the percentage of identical amino acids that are the same in both PB2 sequences). Blue: PB2 protein sequences from avian IAVs. Red: PB2 protein sequences from human IAVs. Green: PB2 protein sequences from mammalian IAVs.


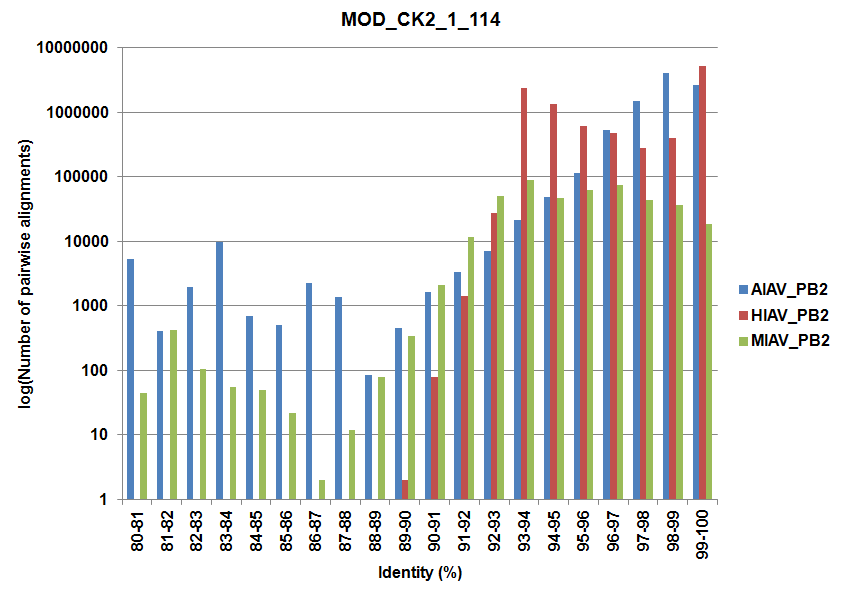


PB2 Identity Distribution 3. The distribution of pairwise alignment identity of PB2 protein sequences which harbor the SLiM MOD_CK2_1_114 from avian, human and mammalian IAVs. The x-axis is the number of pairwise alignments of IAV PB2 protein sequences. The y-axis is the identity of pairwise alignment (the percentage of identical amino acids that are the same in both PB2 sequences). Blue: PB2 protein sequences from avian IAVs. Red: PB2 protein sequences from human IAVs. Green: PB2 protein sequences from mammalian IAVs.


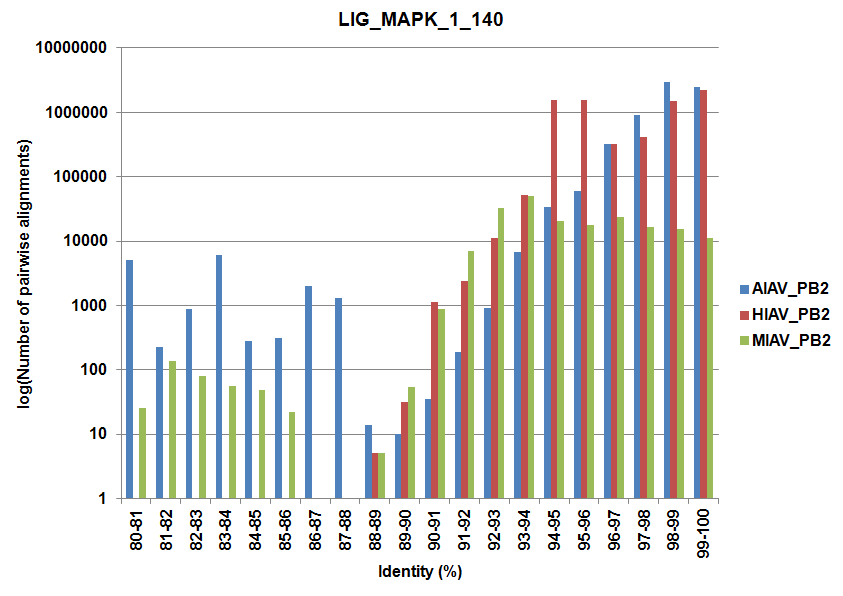


PB2 Identity Distribution 4. The distribution of pairwise alignment identity of PB2 protein sequences which harbor the SLiM LIG_MAPK_1_140 from avian, human and mammalian IAVs. The x-axis is the number of pairwise alignments of IAV PB2 protein sequences. The y-axis is the identity of pairwise alignment (the percentage of identical amino acids that are the same in both PB2 sequences). Blue: PB2 protein sequences from avian IAVs. Red: PB2 protein sequences from human IAVs. Green: PB2 protein sequences from mammalian IAVs.


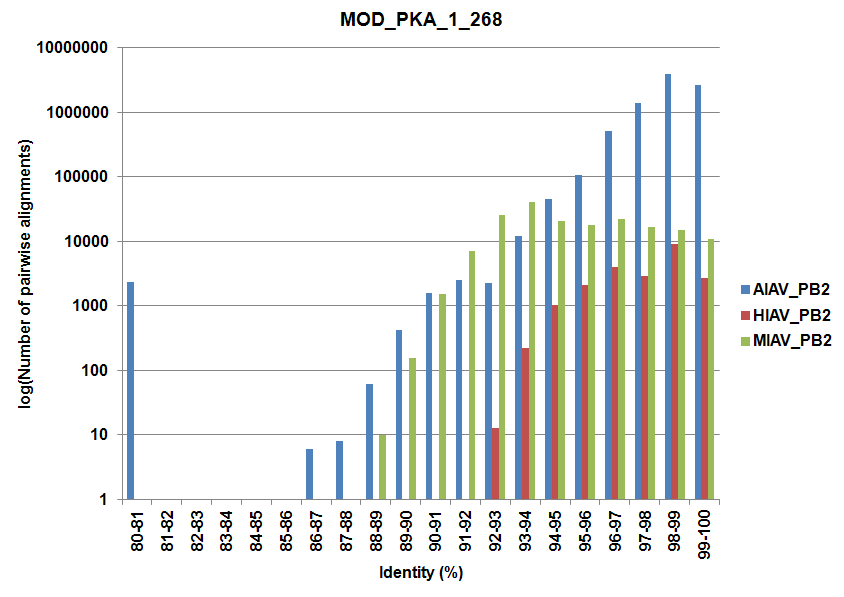


PB2 Identity Distribution 5. The distribution of pairwise alignment identity of PB2 protein sequences which harbor the SLiM MOD_PKA_1_268 from avian, human and mammalian IAVs. The x-axis is the number of pairwise alignments of IAV PB2 protein sequences. The y-axis is the identity of pairwise alignment (the percentage of identical amino acids that are the same in both PB2 sequences). Blue: PB2 protein sequences from avian IAVs. Red: PB2 protein sequences from human IAVs. Green: PB2 protein sequences from mammalian IAVs.


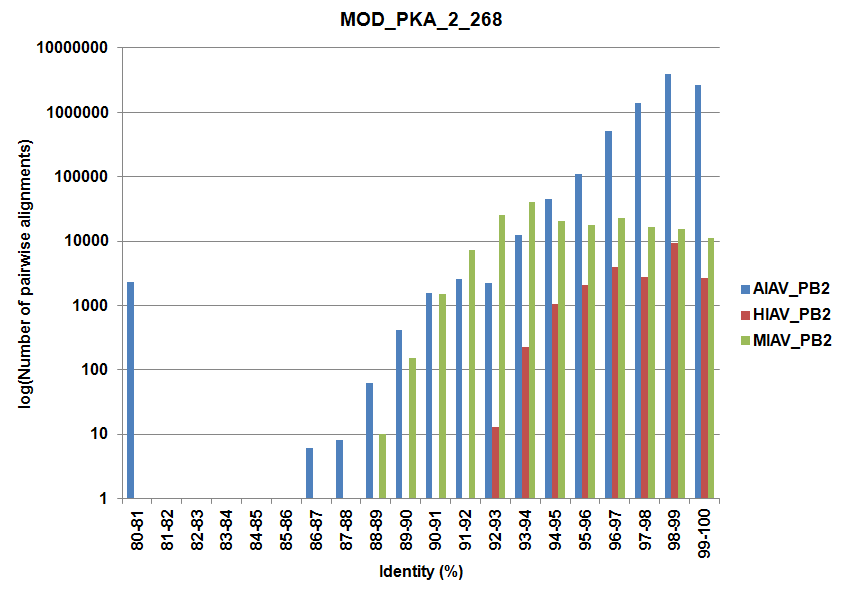


PB2 Identity Distribution 6. The distribution of pairwise alignment identity of PB2 protein sequences which harbor the SLiM MOD_PKA_2_268 from avian, human and mammalian IAVs. The x-axis is the number of pairwise alignments of IAV PB2 protein sequences. The y-axis is the identity of pairwise alignment (the percentage of identical amino acids that are the same in both PB2 sequences). Blue: PB2 protein sequences from avian IAVs. Red: PB2 protein sequences from human IAVs. Green: PB2 protein sequences from mammalian IAVs.


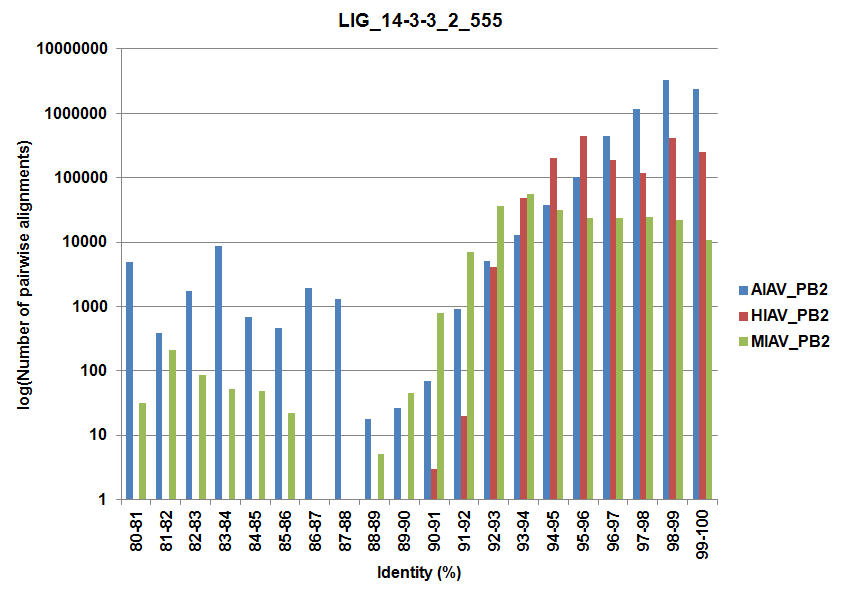


PB2 Identity Distribution 7. The distribution of pairwise alignment identity of PB2 protein sequences which harbor the SLiM LIG_14-3-3_2_555 from avian, human and mammalian IAVs. The x-axis is the number of pairwise alignments of IAV PB2 protein sequences. The y-axis is the identity of pairwise alignment (the percentage of identical amino acids that are the same in both PB2 sequences). Blue: PB2 protein sequences from avian IAVs. Red: PB2 protein sequences from human IAVs. Green: PB2 protein sequences from mammalian IAVs.


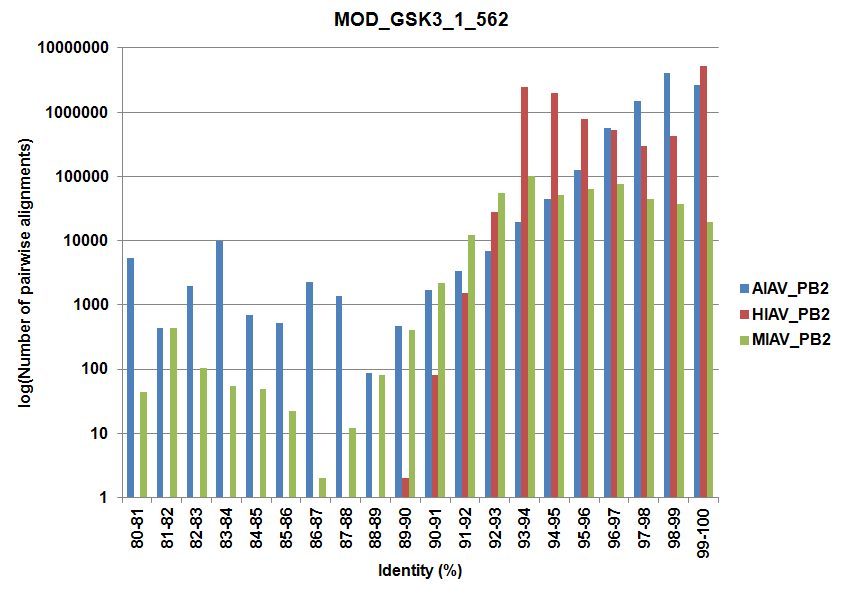


PB2 Identity Distribution 8. The distribution of pairwise alignment identity of PB2 protein sequences which harbor the SLiM MOD_GSK3_1_562 from avian, human and mammalian IAVs. The x-axis is the number of pairwise alignments of IAV PB2 protein sequences. The y-axis is the identity of pairwise alignment (the percentage of identical amino acids that are the same in both PB2 sequences). Blue: PB2 protein sequences from avian IAVs. Red: PB2 protein sequences from human IAVs. Green: PB2 protein sequences from mammalian IAVs.


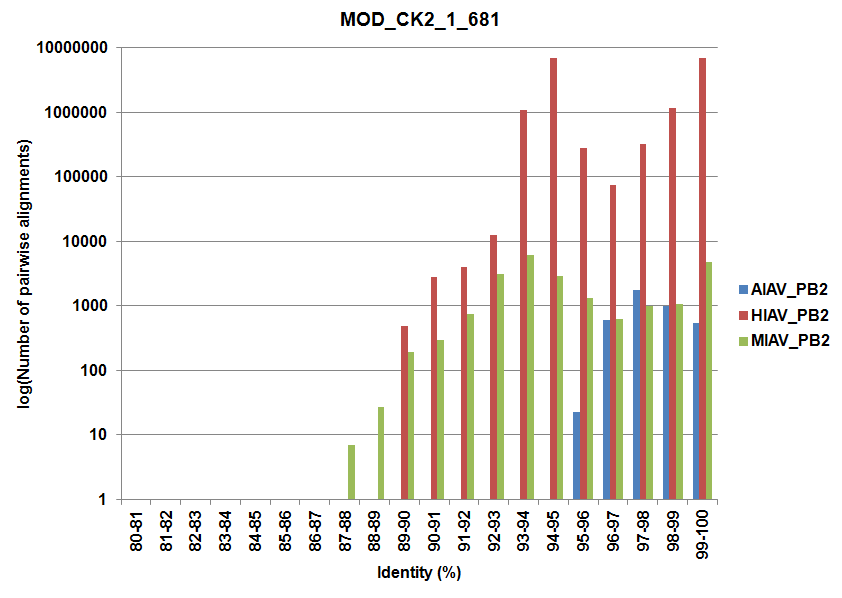


PB2 Identity Distribution 9. The distribution of pairwise alignment identity of PB2 protein sequences which harbor the SLiM MOD_CK2_1_681 from avian, human and mammalian IAVs. The x-axis is the number of pairwise alignments of IAV PB2 protein sequences. The y-axis is the identity of pairwise alignment (the percentage of identical amino acids that are the same in both PB2 sequences). Blue: PB2 protein sequences from avian IAVs. Red: PB2 protein sequences from human IAVs. Green: PB2 protein sequences from mammalian IAVs.


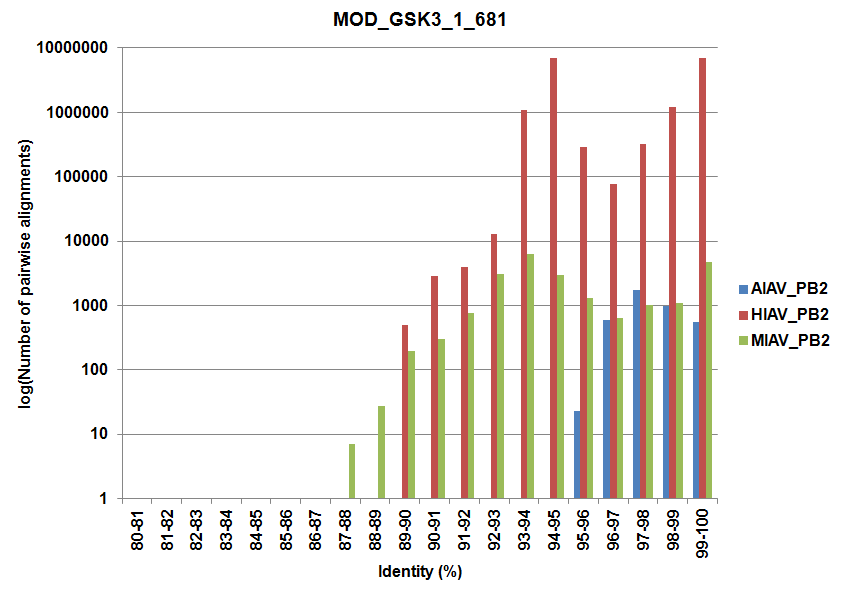


PB2 Identity Distribution 10. The distribution of pairwise alignment identity of PB2 protein sequences which harbor the SLiM MOD_GSK3_1_681 from avian, human and mammalian IAVs. The x-axis is the number of pairwise alignments of IAV PB2 protein sequences. The y-axis is the identity of pairwise alignment (the percentage of identical amino acids that are the same in both PB2 sequences). Blue: PB2 protein sequences from avian IAVs. Red: PB2 protein sequences from human IAVs. Green: PB2 protein sequences from mammalian IAVs.
