# Supplementary material for: A Comparative Study of Short Linear Motif Compositions of the Influenza A Virus Ribonucleoproteins
Source: PLoS One. 2012 Jun 8;7(6):e38637. doi: 10.1371/journal.pone.0038637 (PMC3371030; doi:10.1371/journal.pone.0038637)
Supplement: Information S21 — Highly conserved SLiMs in IAV NP proteins. (DOC) [file pone.0038637.s021.doc]

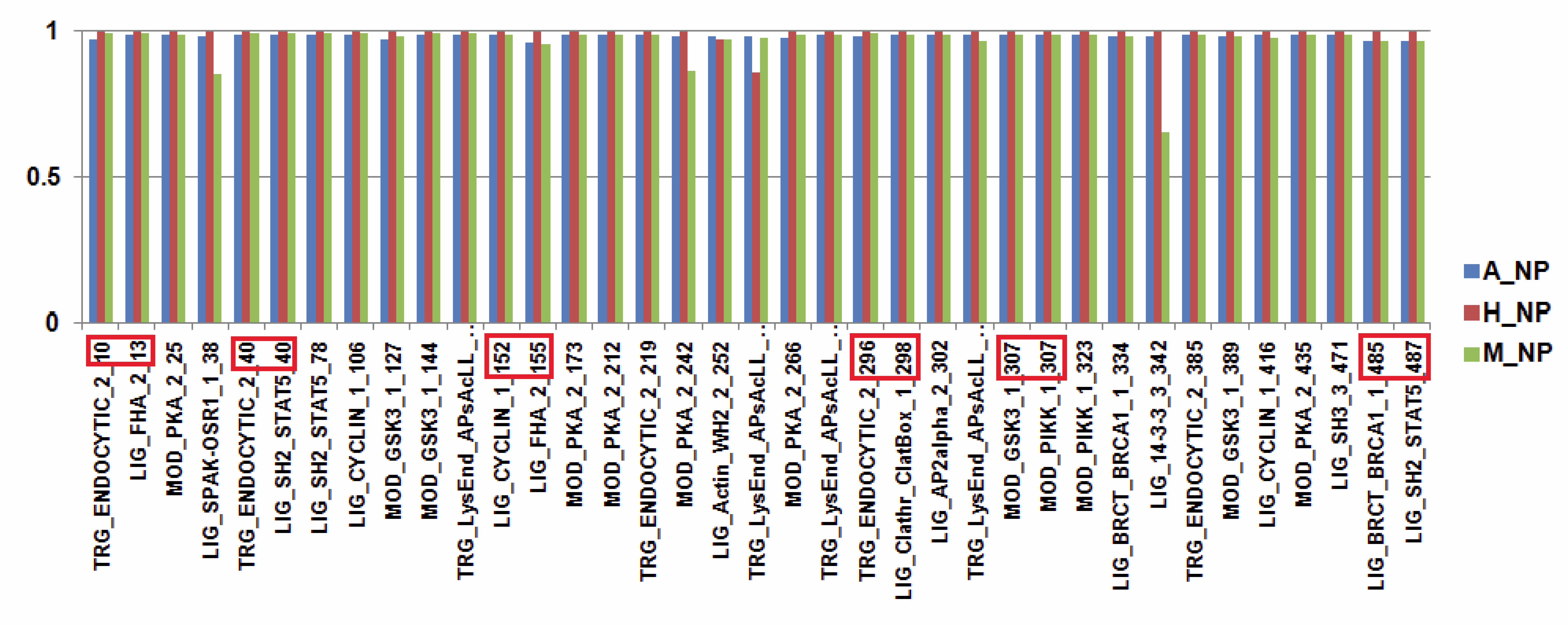


## Figure S21. Highly conserved SLiMs in IAV NP proteins.

The Y-axis indicates the occurrence of each identified SLiM. The X-axis indicates the name and position of each identified SLiM in the NP proteins. For example, “LIG_FHA_2” in “LIG_FHA_2_13” is the name of the SLiM, and 13 is the amino acid position where the SLiM starts. The red rectangles indicate overlapping SLiMs. A_NP, H_NP and M_NP indicate the NP proteins from avian, human and IAV, respectively.
