# Supplementary material for: A Comparative Study of Short Linear Motif Compositions of the Influenza A Virus Ribonucleoproteins
Source: PLoS One. 2012 Jun 8;7(6):e38637. doi: 10.1371/journal.pone.0038637 (PMC3371030; doi:10.1371/journal.pone.0038637)
Supplement: Information S22 — The identity distributions of SLiMs from IAV PB2 proteins that have differential occurrences in IAVs from different hosts. (DOC) [file pone.0038637.s022.doc]

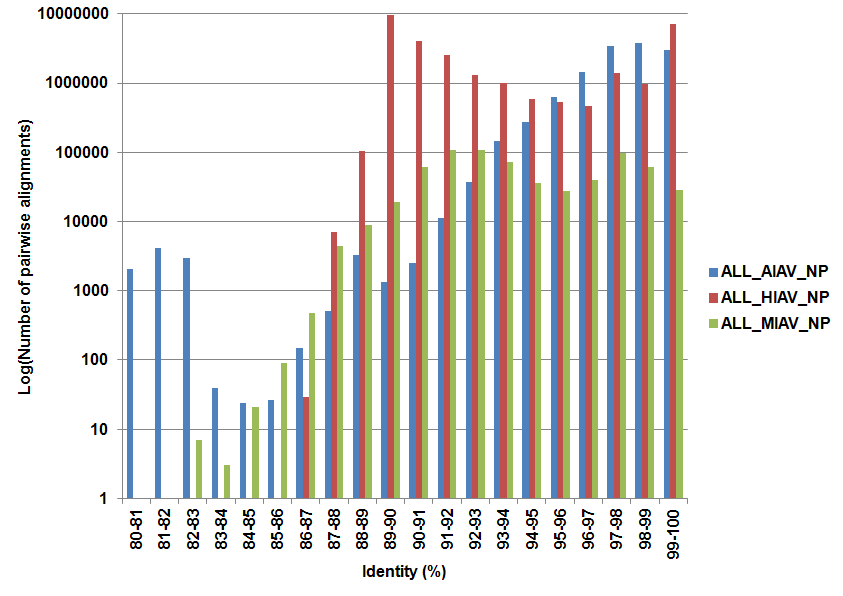


NP Identity Distribution 1. The distribution of pairwise alignment identity of all NP protein sequences from avian, human and mammalian IAVs. The x-axis is the number of pairwise alignments of IAV NP protein sequences. The y-axis is the identity of pairwise alignment (the percentage of identical amino acids that are the same in both NP sequences). Blue: NP protein sequences from avian IAVs. Red: NP protein sequences from human IAVs. Green: NP protein sequences from mammalian IAVs.


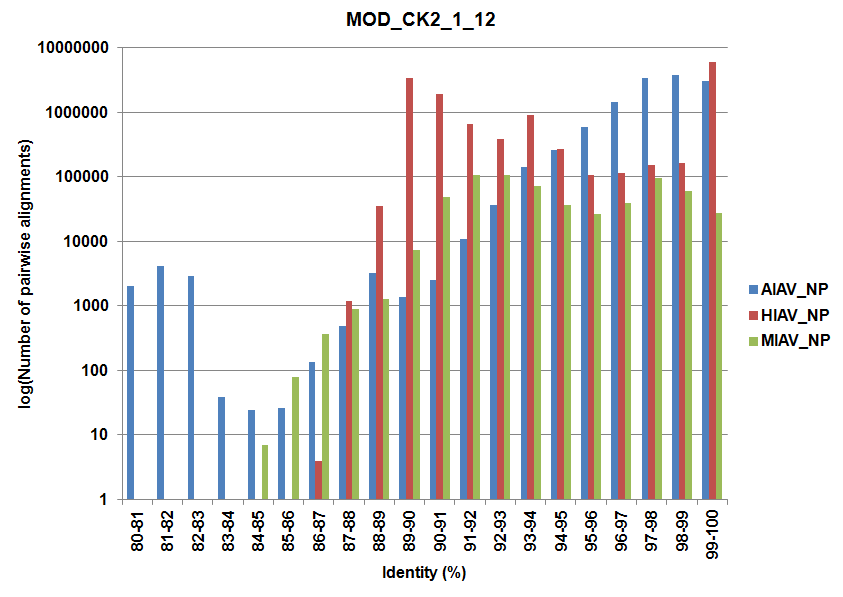


NP Identity Distribution 2. The distribution of pairwise alignment identity of NP protein sequences which harbor the SLiM MOD_CK2_1_12 from avian, human and mammalian IAVs. The x-axis is the number of pairwise alignments of IAV NP protein sequences. The y-axis is the identity of pairwise alignment (the percentage of identical amino acids that are the same in both NP sequences). Blue: NP protein sequences from avian IAVs. Red: NP protein sequences from human IAVs. Green: NP protein sequences from mammalian IAVs.


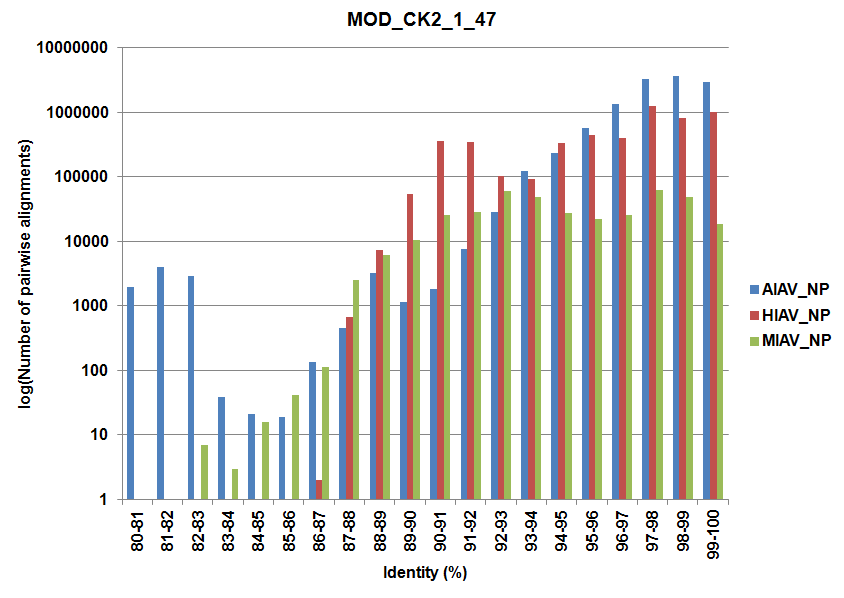


NP Identity Distribution 3. The distribution of pairwise alignment identity of NP protein sequences which harbor the SLiM MOD_CK2_1_47 from avian, human and mammalian IAVs. The x-axis is the number of pairwise alignments of IAV NP protein sequences. The y-axis is the identity of pairwise alignment (the percentage of identical amino acids that are the same in both NP sequences). Blue: NP protein sequences from avian IAVs. Red: NP protein sequences from human IAVs. Green: NP protein sequences from mammalian IAVs.


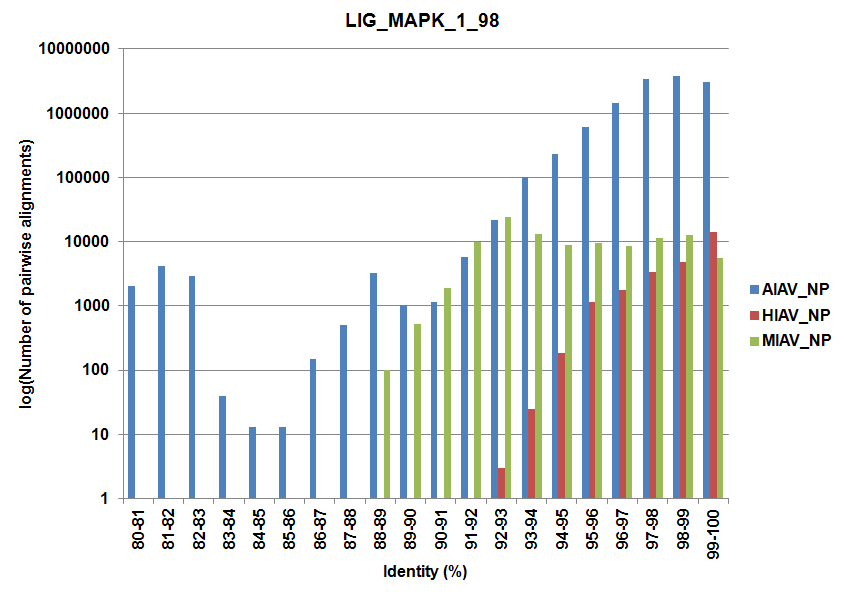


NP Identity Distribution 4. The distribution of pairwise alignment identity of NP protein sequences which harbor the SLiM LIG_MAPK_1_98 from avian, human and mammalian IAVs. The x-axis is the number of pairwise alignments of IAV NP protein sequences. The y-axis is the identity of pairwise alignment (the percentage of identical amino acids that are the same in both NP sequences). Blue: NP protein sequences from avian IAVs. Red: NP protein sequences from human IAVs. Green: NP protein sequences from mammalian IAVs.


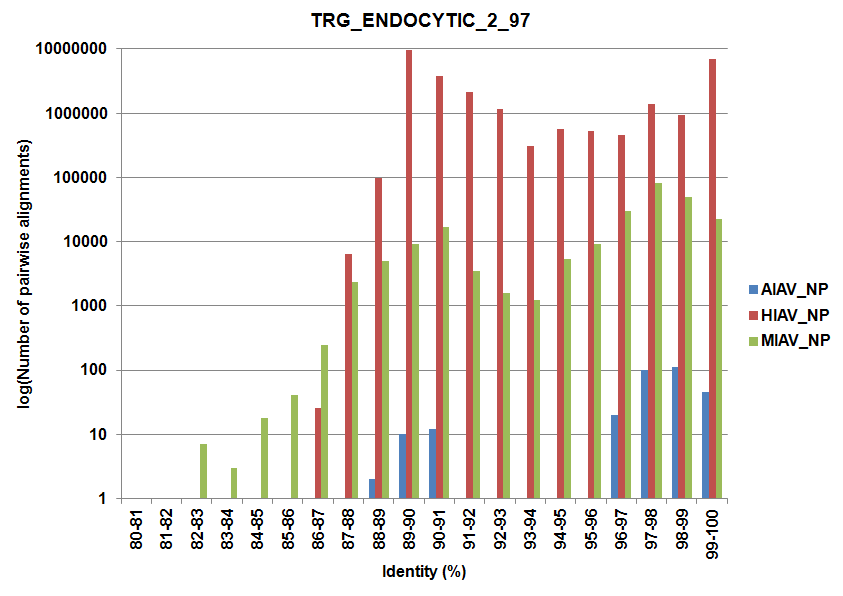


NP Identity Distribution 5. The distribution of pairwise alignment identity of NP protein sequences which harbor the SLiM TRG_ENDOCYTIC_2_97 from avian, human and mammalian IAVs. The x-axis is the number of pairwise alignments of IAV NP protein sequences. The y-axis is the identity of pairwise alignment (the percentage of identical amino acids that are the same in both NP sequences). Blue: NP protein sequences from avian IAVs. Red: NP protein sequences from human IAVs. Green: NP protein sequences from mammalian IAVs.


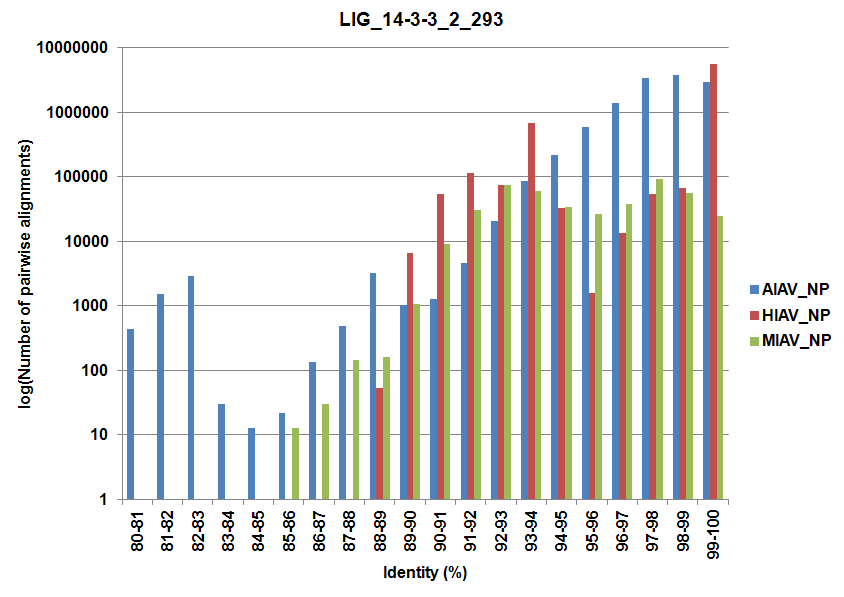


NP Identity Distribution 6. The distribution of pairwise alignment identity of NP protein sequences which harbor the SLiM LIG_14-3-3_2_293 from avian, human and mammalian IAVs. The x-axis is the number of pairwise alignments of IAV NP protein sequences. The y-axis is the identity of pairwise alignment (the percentage of identical amino acids that are the same in both NP sequences). Blue: NP protein sequences from avian IAVs. Red: NP protein sequences from human IAVs. Green: NP protein sequences from mammalian IAVs.


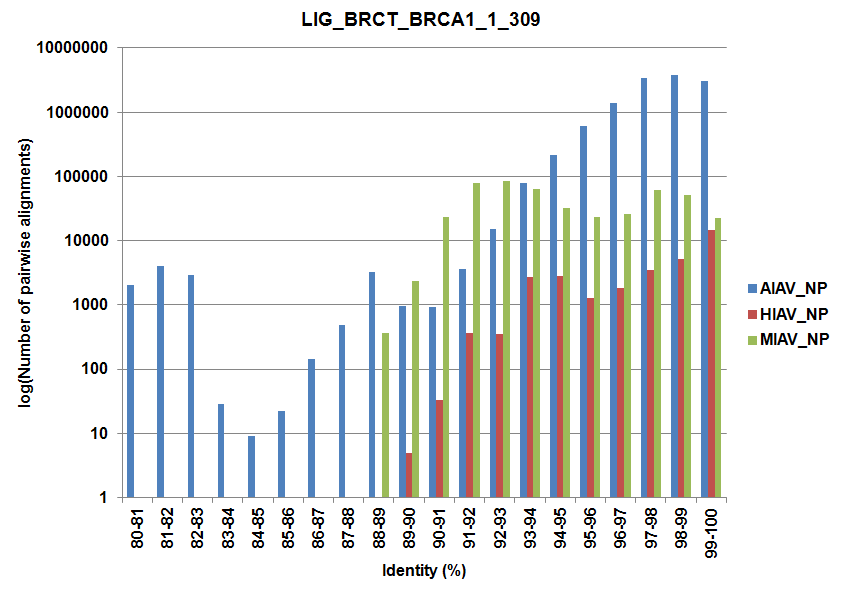


NP Identity Distribution 7. The distribution of pairwise alignment identity of NP protein sequences which harbor the SLiM LIG_BRCT_BRCA1_1_309 from avian, human and mammalian IAVs. The x-axis is the number of pairwise alignments of IAV NP protein sequences. The y-axis is the identity of pairwise alignment (the percentage of identical amino acids that are the same in both NP sequences). Blue: NP protein sequences from avian IAVs. Red: NP protein sequences from human IAVs. Green: NP protein sequences from mammalian IAVs.


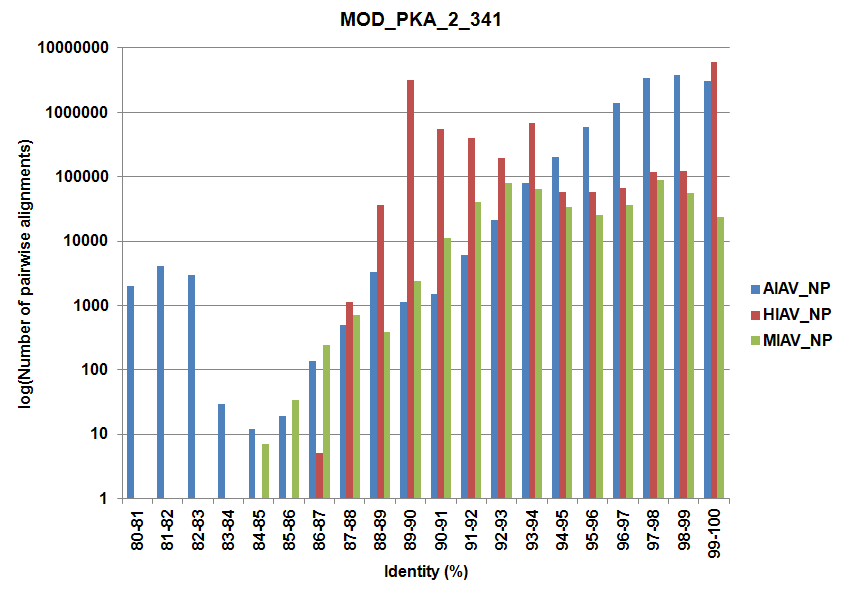


NP Identity Distribution 8. The distribution of pairwise alignment identity of NP protein sequences which harbor the SLiM MOD_PKA_2_341 from avian, human and mammalian IAVs. The x-axis is the number of pairwise alignments of IAV NP protein sequences. The y-axis is the identity of pairwise alignment (the percentage of identical amino acids that are the same in both NP sequences). Blue: NP protein sequences from avian IAVs. Red: NP protein sequences from human IAVs. Green: NP protein sequences from mammalian IAVs.


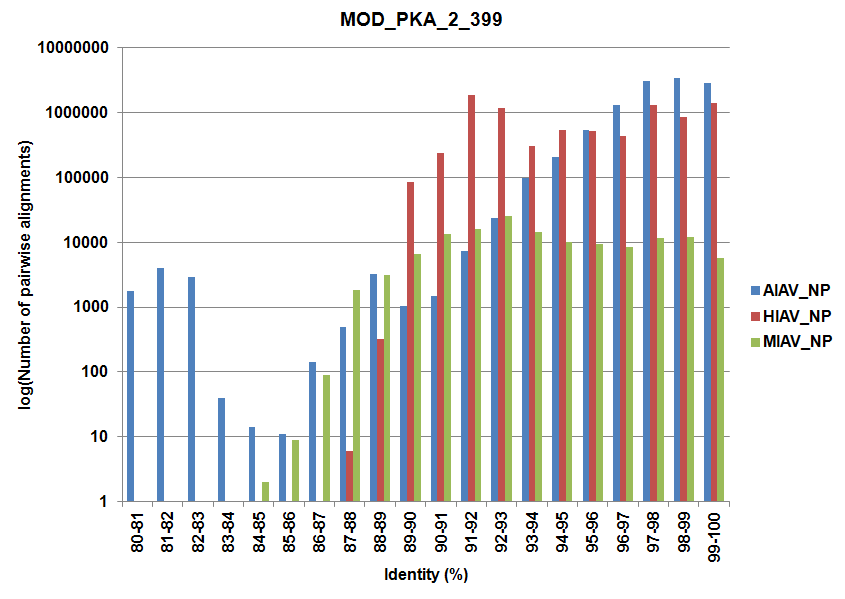


NP Identity Distribution 9. The distribution of pairwise alignment identity of NP protein sequences which harbor the SLiM MOD_PKA_2_399 from avian, human and mammalian IAVs. The x-axis is the number of pairwise alignments of IAV NP protein sequences. The y-axis is the identity of pairwise alignment (the percentage of identical amino acids that are the same in both NP sequences). Blue: NP protein sequences from avian IAVs. Red: NP protein sequences from human IAVs. Green: NP protein sequences from mammalian IAVs.


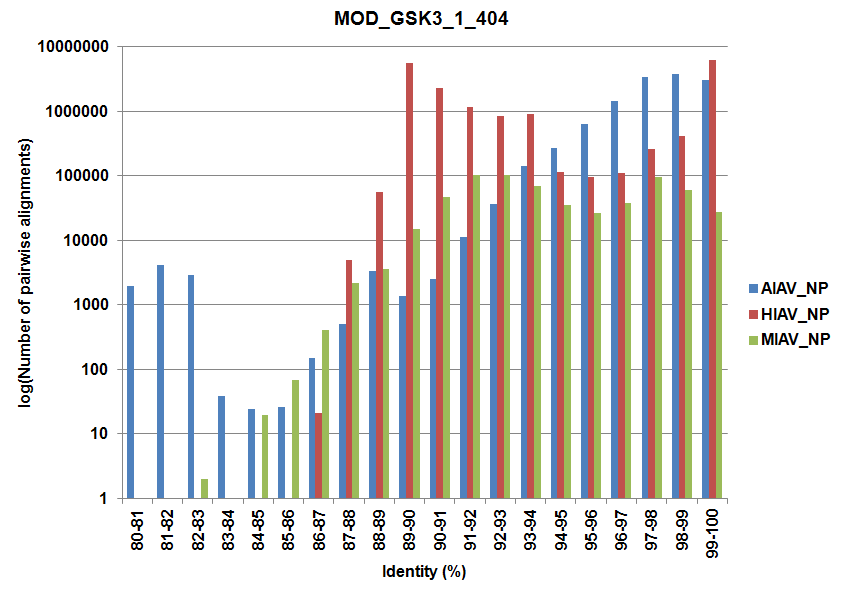


NP Identity Distribution 10. The distribution of pairwise alignment identity of NP protein sequences which harbor the SLiM MOD_GSK3_1_404 from avian, human and mammalian IAVs. The x-axis is the number of pairwise alignments of IAV NP protein sequences. The y-axis is the identity of pairwise alignment (the percentage of identical amino acids that are the same in both NP sequences). Blue: NP protein sequences from avian IAVs. Red: NP protein sequences from human IAVs. Green: NP protein sequences from mammalian IAVs.


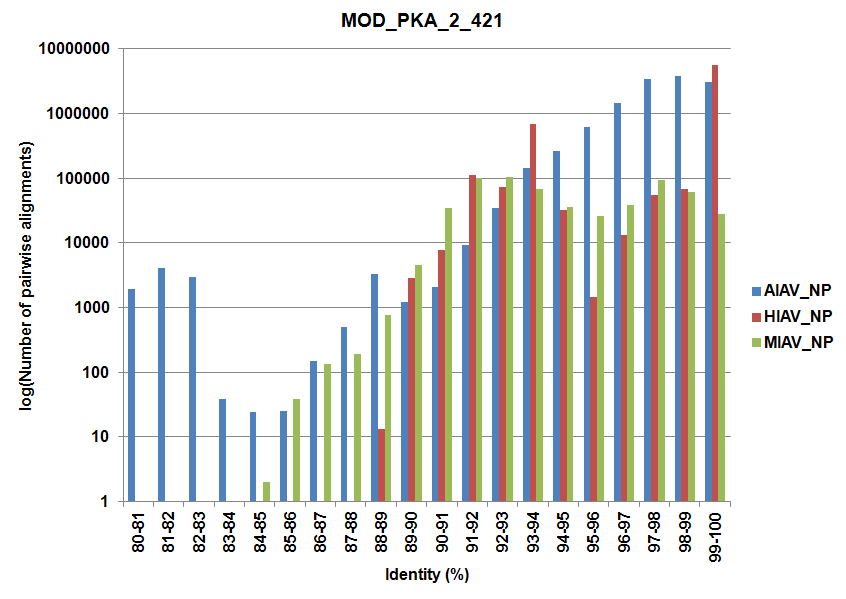


NP Identity Distribution 11. The distribution of pairwise alignment identity of NP protein sequences which harbor the SLiM MOD_PKA_2_421 from avian, human and mammalian IAVs. The x-axis is the number of pairwise alignments of IAV NP protein sequences. The y-axis is the identity of pairwise alignment (the percentage of identical amino acids that are the same in both NP sequences). Blue: NP protein sequences from avian IAVs. Red: NP protein sequences from human IAVs. Green: NP protein sequences from mammalian IAVs.


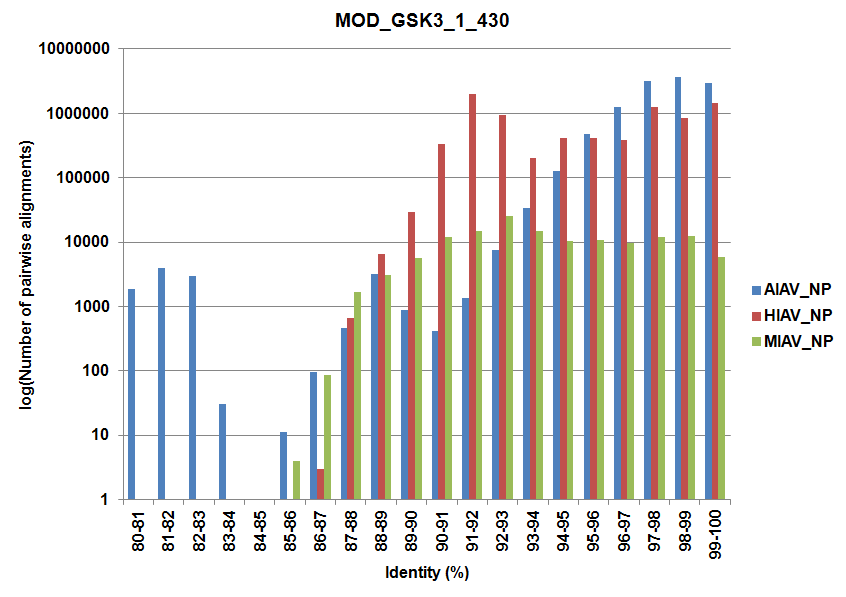


NP Identity Distribution 12. The distribution of pairwise alignment identity of NP protein sequences which harbor the SLiM MOD_GSK3_1_430 from avian, human and mammalian IAVs. The x-axis is the number of pairwise alignments of IAV NP protein sequences. The y-axis is the identity of pairwise alignment (the percentage of identical amino acids that are the same in both NP sequences). Blue: NP protein sequences from avian IAVs. Red: NP protein sequences from human IAVs. Green: NP protein sequences from mammalian IAVs.


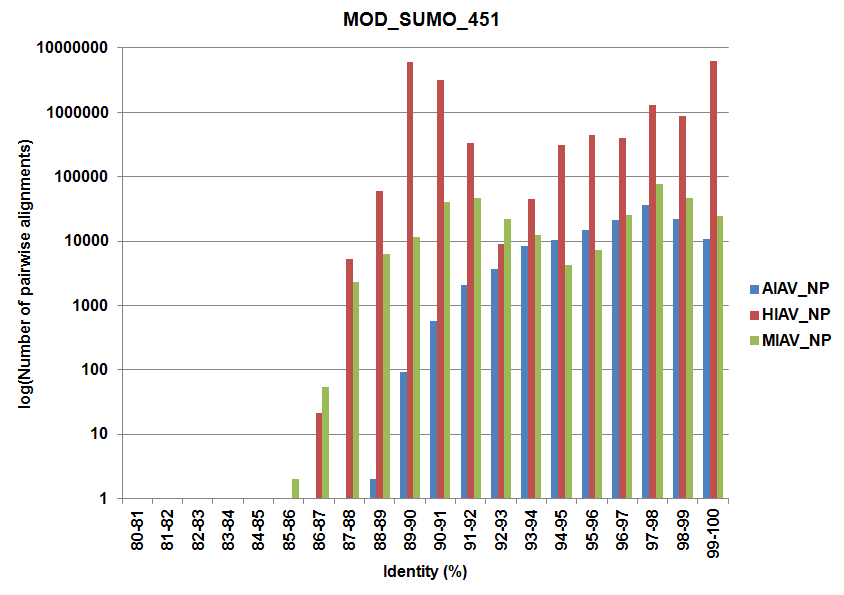


NP Identity Distribution 13. The distribution of pairwise alignment identity of NP protein sequences which harbor the SLiM MOD_SUMO_451 from avian, human and mammalian IAVs. The x-axis is the number of pairwise alignments of IAV NP protein sequences. The y-axis is the identity of pairwise alignment (the percentage of identical amino acids that are the same in both NP sequences). Blue: NP protein sequences from avian IAVs. Red: NP protein sequences from human IAVs. Green: NP protein sequences from mammalian IAVs.


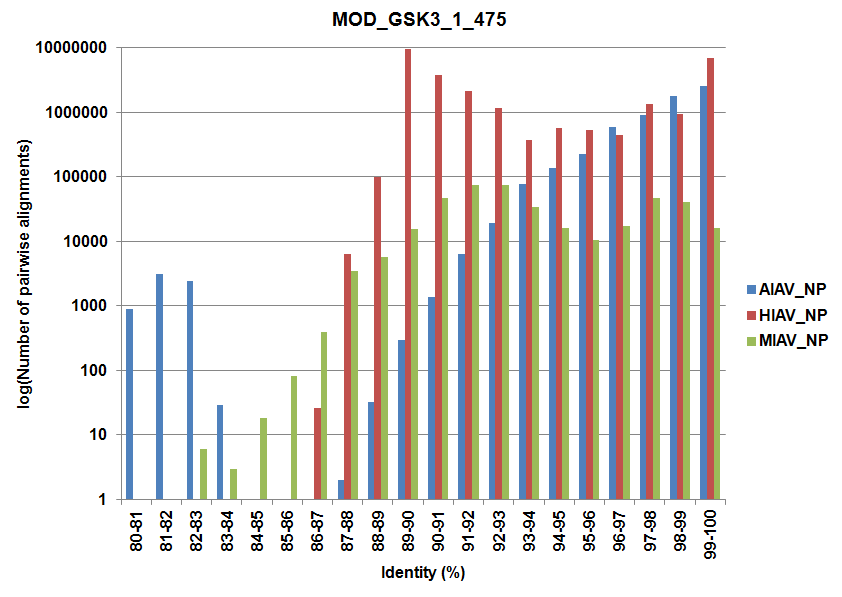


NP Identity Distribution 14. The distribution of pairwise alignment identity of NP protein sequences which harbor the SLiM MOD_GSK3_1_475 from avian, human and mammalian IAVs. The x-axis is the number of pairwise alignments of IAV NP protein sequences. The y-axis is the identity of pairwise alignment (the percentage of identical amino acids that are the same in both NP sequences). Blue: NP protein sequences from avian IAVs. Red: NP protein sequences from human IAVs. Green: NP protein sequences from mammalian IAVs.
